# The unique stem cell system of the immortal larva of the human parasite *Echinococcus multilocularis*

**DOI:** 10.1186/2041-9139-5-10

**Published:** 2014-03-06

**Authors:** Uriel Koziol, Theresa Rauschendorfer, Luis Zanon Rodríguez, Georg Krohne, Klaus Brehm

**Affiliations:** 1 Institute of Hygiene and Microbiology, University of Würzburg, Josef-Schneider-Strasse 2, D-97080 Würzburg, Germany; 2 Department of Electron Microscopy, University of Würzburg, Biocenter, D-97078 Würzburg, Germany; 3 Facultad de Ciencias, Sección Bioquímica y Biología Molecular, Universidad de la República, Iguá 4225, CP 11400 Montevideo, Uruguay

**Keywords:** Cestoda, Echinococcus, Neoblast, Germinative cell, Stem cell, Nanos, Argonaute, Mucin, Alkaline phosphatase

## Abstract

**Background:**

It is believed that in tapeworms a separate population of undifferentiated cells, the germinative cells, is the only source of cell proliferation throughout the life cycle (similar to the neoblasts of free living flatworms). In *Echinococcus multilocularis*, the metacestode larval stage has a unique development, growing continuously like a mass of vesicles that infiltrate the tissues of the intermediate host, generating multiple protoscoleces by asexual budding. This unique proliferation potential indicates the existence of stem cells that are totipotent and have the ability for extensive self-renewal.

**Results:**

We show that only the germinative cells proliferate in the larval vesicles and in primary cell cultures that undergo complete vesicle regeneration, by using a combination of morphological criteria and by developing molecular markers of differentiated cell types. The germinative cells are homogeneous in morphology but heterogeneous at the molecular level, since only sub-populations express homologs of the post-transcriptional regulators *nanos* and *argonaute*. Important differences are observed between the expression patterns of selected neoblast marker genes of other flatworms and the *E. multilocularis* germinative cells, including widespread expression in *E. multilocularis* of some genes that are neoblast-specific in planarians. Hydroxyurea treatment results in the depletion of germinative cells in larval vesicles, and after recovery following hydroxyurea treatment, surviving proliferating cells grow as patches that suggest extensive self-renewal potential for individual germinative cells.

**Conclusions:**

In *E. multilocularis* metacestodes, the germinative cells are the only proliferating cells, presumably driving the continuous growth of the larval vesicles. However, the existence of sub-populations of the germinative cells is strongly supported by our data. Although the germinative cells are very similar to the neoblasts of other flatworms in function and in undifferentiated morphology, their unique gene expression pattern and the evolutionary loss of conserved stem cells regulators suggest that important differences in their physiology exist, which could be related to the unique biology of *E. multilocularis* larvae.

## Background

The Platyhelminthes (flatworms) comprise a highly diverse phylum in terms of morphology, embryology, life-cycle complexity and capacity for regeneration and asexual reproduction [1-4]. However, they are united by having a unique population of undifferentiated stem cells, commonly known as ‘neoblasts’ [5,6]. It is thought that neoblasts represent the only proliferative cell population, and are therefore the source of new cells for normal tissue turnover, growth and regeneration.

The characterization of neoblasts has been most extensive for free living flatworms, especially for planarians. Planarian neoblasts have been shown to be truly pluripotent [7], and are essential for planarian regeneration [8]. Classical ultrastructural studies in planarians described the neoblasts as small, round cells with a large nucleus containing little heterochromatin and a large nucleolus, with scant cytoplasm containing mitochondria, abundant free ribosomes and few other organelles [9,10]. Furthermore, they possess cytoplasmic electron-dense ribonucleoprotein (RNP) granules called chromatoid bodies, which are molecularly and morphologically similar to the well known germ granules present in the germ cells of many animals. Germ granules are thought to function as centers for post-transcriptional regulation of mRNA, similar to other RNP bodies in somatic cells [11,12]. Many studies have shown that genes involved in post-transcriptional regulation and chromatin modification are highly upregulated in neoblasts [13-18]. These include genes that are typically considered markers of germ cells in other model animals, such as the DEAD box RNA helicase *vasa* and the Argonaute family gene *piwi*[11]. This expression of germ line markers in somatic multipotent stem cells has also been found in other animal lineages, and has been interpreted as part of a multipotency program conserved between the germ line and multipotent somatic stem cells [19]. The development of molecular markers has further shown that the neoblasts are actually heterogeneous at the molecular level [9,10].

The main groups of parasitic flatworms, including cestodes, trematodes and monogeneans, form the monophyletic clade Neodermata [4,20]. In cestodes, classical studies have evidenced a population of undifferentiated stem cell similar to the neoblasts, which are denominated the germinative cells [21-28]. However, unlike for planarian neoblasts, chromatoid bodies have never been described for the germinative cells. Germinative cells are thought to drive the development throughout the cestode life cycle. In the ‘typical’ cestode life cycle, the oncosphere (first larval stage) is a highly reduced organism that has a small population of set-aside germinative cells. Once the oncosphere infects the intermediate host, it develops into the metacestode (second larval stage), and it is thought that only the germinative cells contribute to this metamorphosis [29]. Usually, the metacestode is similar to a ‘juvenile’ tapeworm, containing the scolex (head) with the attachment organs, but lacking the reproductive systems. Finally, the intermediate host containing the metacestode is ingested by the definitive host, and the metacestode develops into an adult in the intestine. New segments, each containing a complete set of male and female reproductive systems, are generated continuously from the proliferative region of the neck, behind the scolex, and produce oncospheres by sexual reproduction. In the neck region of segmenting cestodes, the proliferating germinative cells are localized close to the inner muscle layer, and have been shown to be the only proliferating cell type [22-24,27,30].

**Figure 1:**
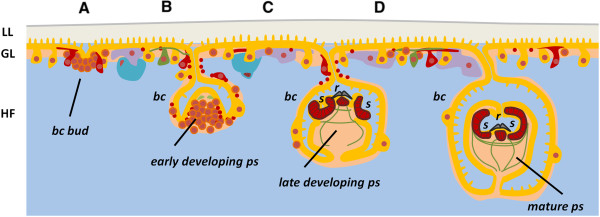
**Schematic drawing showing the general organization and development of *E. multilocularis *metacestodes. (A)** Early brood capsule bud. **(B)** Brood capsule with protoscolex bud. **(C)** Brood capsule with protoscolex in late development. **(D)** Brood capsule with invaginated protoscolex. The syncitial tegument is shown in orange, the germinative cells in brown, glycogen/lipid storage cells in violet, calcareous corpuscle cells in light blue, nerve cells in green and muscle cells and fibers in red. bc, brood capsule; GL, germinal layer; HF, hydatid fluid; LL, laminated layer; ps, protoscolex; r, rostellum; s, sucker.

The metacestode stage of *Echinococcus multilocularis* is atypical in its development and morphology [31-33]. After the oncosphere is ingested by the intermediate host (diverse rodents, but also accidentally by humans) it develops in the liver as a labyrinth of vesicles, which grow cancer-like and infiltrate the tissue of the host, forming new vesicles and even metastases. The metacestode growth causes the disease alveolar echinococcosis, one of the most dangerous zoonoses of the Northern Hemisphere [33]. The metacestode vesicles comprise a thin layer of tissue (the germinal layer) covered by a syncitialtegument that secretes an acellular, carbohydrate-rich external layer (the laminated layer) (Figure 1). The remaining volume of the vesicles is filled with fluid (hydatid fluid). Within the germinal layer, thickenings (buds) occur that invaginate into the vesicle, resulting in the formation of brood capsules (Figure 1A). Within the brood capsules, a new budding process occurs, that results in the formation of protoscoleces, the infective form for the definitive host (Figure 1B-C). The protoscolex already resembles the anterior region of the adult form, with a scolex that lays invaginated within a small posterior body (Figure 1D). After ingestion of the protoscolex by the definitive host (canids), it evaginates its scolex, attaches to the intestine and develops into the adult tapeworm [33].

The metacestode tissue can be maintained and will grow indefinitely in intermediate hosts by serial passage, and is in this sense ‘immortal’ [34,35]. Recently, we have developed methods for the axenic *in vitro* maintenance of metacestode vesicles, and for primary cell cultures that result in complete regeneration of metacestode vesicles [36]. These methods allow for *in vitro* analysis of development in *Echinococcus* metacestodes, and show that at least at a population level, the primary cell preparations are multipotent. Classical ultrastructural studies in *E. multilocularis* and the related *Echinococcus granulosus* demonstrated the existence of germinative cells in the germinal layer, which proliferate and accumulate during brood capsule and protoscolex development [28]. This accumulation of proliferating cells in the developing protoscolex was confirmed by labeling with radioactive thymidine [37]. Nothing is known to date about gene expression in cestode germinative cells, but the genome sequencing project of *E. multilocularis* demonstrated the lack of *vasa* and *piwi* orthologs, suggesting fundamental differences between germinative cells and planarian neoblasts [38]. Differentiated cell types have also been described in the germinal layer, including tegumental cells (the cell bodies of the tegumental syncitium, which are connected to the overlying syncitial tegument by cytoplasmatic bridges), muscle cells, glycogen/lipid storing cells, and recently, nerve cells [28,39,40].

In this work, we characterize the germinative cells in the metacestodes and in primary cell cultures as the only proliferating cells, driving metacestode growth and regeneration. By developing methods for analyzing gene expression with cellular resolution in *E. multilocularis*, we show that differentiated cell types do not proliferate, and that the germinative cells are heterogeneous at the molecular level, showing in addition several differences with the neoblasts from other flatworms. Finally, by analyzing the response of the metacestodes after partial germinative cell depletion, we provide evidence that indicates extensive self-renewal capabilities for individual germinative cells.

## Methods

### Parasite material, culture and primary cell preparation

Parasite isolates were maintained by serial intraperitoneal passage in *Meriones unguiculatus* as previously described [34]. Unless otherwise stated, all experiments were performed on *in vitro* cultured metacestodes. Standard *in vitro* culture of metacestodes was done in co-culture with rat Reuber hepatoma feeder cells, and primary cell preparations were performed and cultured in cDMEM-A pre-conditioned medium essentially as previously described [34], with the following modifications: 1) cells were detached from the metacestode tissue with a single treatment of 20 minutes with trypsin/ethylenediaminetetraacetic acid (EDTA) and 2) primary cells were cultured in cDMEM-A instead of hydatid fluid.

For primary cell cultures, isolate H95 [41], which has been passaged for 18 years and has developed a strong defect in protoscolex formation was used. For other experiments, more recent isolates were used, obtained from accidental infections of Old World Monkeys in a breeding exclosure [42]. *Dugesia tahitiensis* ([43], obtained from Bernhard Egger) was the planarian species used for immunohistofluorescence.

### Ethical approval

All experiments were carried out in accordance with European and German regulations on the protection of animals (*Tierschutzgesetz*). Ethical approval of the study was obtained from the local ethics committee of the government of Lower Franconia (55.2-2531.01-31/10).

### EdU labeling and detection

For short term labeling, 50 μM of 5-ethynyl-2′-deoxyuridine (EdU, Life Technologies, Darmstadt, Germany) was added to the media and the material was incubated for five hours. For continuous labeling, 1 μM EdU was used for up to 14 days. The length of incubation and the EdU concentrations were determined after varying the parameters in preliminary experiments (see the main text). Before fixation, metacestode vesicles were gently opened using a syringe tip in order to allow the entry of the fixative and other reagents during detection procedures. Samples were fixed in 4% paraformaldehyde prepared in PBS (PFA-PBS) for one hour at room temperature, and processed for detection of EdU in paraplast sections. Detection was performed with the Click-iT® EdU Alexa Fluor® 555 Imaging Kit (Life Technologies, Darmstadt, Germany) as described by the manufacturer for sections. Whole-mount detection was performed by a modified protocol in which all steps were doubled in length and the washes were increased in number. For double labeling, EdU detection was always performed after the immunohistofluorescence or *in situ* hybridization protocols.

### Tissue maceration and staining of cell suspensions

Cell suspensions were prepared by a modification of the method of David [44]. Metacestode vesicles were opened and washed in PBS and placed in maceration solution (13:1:1 distilled water: glacial acetic acid: glycerol, 100 μl of solution per vesicle). Primary cell aggregates (from one well of a six-well plate, after two days of culture) were washed in PBS, allowed to settle and placed in 500 μl of maceration solution. Both kinds of samples were pipetted up and down with a p1000 pipette, and placed overnight at 4°C. The next day they were once again disaggregated by pipetting, diluted to 1:10 with maceration solution, and 10 μl were spotted on SuperFrost slides (Thermo Scientific). The slides were dried overnight at room temperature and stained by either of these procedures:

A) PI plus DAPI: after washing the slides with PBS plus 0.05% Triton X-100, the slides were stained successively with DAPI (1 ug/ml in PBS) and PI (2.5 ug/ml in PBS), washed twice with PBS and mounted with Fluoprep (bioMérieux).

B) WCS plus DAPI: the Cellomics™ WCS Green (Thermo Scientific) was used as instructed by the manufacturer, followed by DAPI staining, washing and Fluoprep mounting. When done in combination, EdU detection was performed first, followed by WCS plus DAPI staining.

C) Nile red plus DAPI: after washing with PBS, the slides were stained with Nile Red [45] (Sigma-Aldrich, Hamburg, Germany; 100 ng/ml in PBS from a 4.2 mg/ml stock in acetone) followed by DAPI staining, washing, and Fluoprep mounting (Biomerieux, Nürtingen, Germany). Imaging was performed with the rhodamine channel of the Zeiss Axio Imager.Z1 microscope (Zeiss, Hamburg, Germany).

### Hydroxyurea treatment and X-ray irradiation

Metacestode vesicles were cultured in axenic, pre-conditioned cDMEM-A medium [34] with a nitrogen gas phase (40 vesicles in 5 ml of medium in 25 cm^2^ cell culture flasks, vertically positioned). HU was added to a final concentration of 40 mM from a 2 M stock (dissolved in medium), whereas only medium was added to controls. HU was added daily to the medium since it is not stable in solution at temperatures *circa* 37°C [46], and the medium was replaced every two days. After seven days of treatment, the vesicles were washed extensively and transferred to HU-free medium. Some vesicles were fixed immediately for immunohistofluorescence and whole-mount *in situ* hybridization. The remaining vesicles were kept in HU-free medium, taking samples for EdU labeling after 1, 4, 9 and 22 days.

Determination of BrdU incorporation in primary cells after HU treatment was done with the Cell Proliferation Elisa, BrdU (Colorimetric) Kit (Roche). Briefly, primary cells were cultured in the presence of 0, 10 or 40 mM HU for 40 hours, after which half of the medium was replaced with fresh medium containing HU and BrdU (10 μM, final concentration). The cells were cultured for four hours and processed for detection as indicated by the manufacturer. For studying the effect of HU on regeneration, primary cells were cultured with 0, 10 or 40 mM HU, changing the medium and HU every 48 to 72 hours. After three weeks, the number of newly formed vesicles was counted.

For X-ray irradiation, a 150 Gy dose was applied to metacestodes with a Faxitron CP160 source (Faxitron, Much, Germany). Vesicles were then set back into axenic culture, taking samples for EdU labeling after 2, 7, 20 and 48 days. Surviving metacestodes were defined as vesicles being able to maintain turgency and with an apparently intact germinal layer as seen under a dissecting microscope.

### PCR, RT-PCR and molecular cloning

For RT-PCR, RNA was extracted with Tri-Reagent (5 PRIME) and 700 ng of total RNA were used for cDNA synthesis using PrimeScript reverse transcriptase (Takara). For the analysis of genes lacking introns, RNA was previously treated with RQ1 DNase (Promega, 2 units/μg for one hour) and mock controls with no reverse transcriptase were done in parallel to ensure that no amplification was obtained from contaminating genomic DNA. For genes with introns, primers were always designed in two separate exons. A list of primers and annealing temperatures, together with the *E. multilocularis* GeneDB codes (http://www.genedb.org/Homepage/Emultilocularis), is included as supplementary material for all genes (Additional file 1).

For semi-quantitative RT-PCR, ten-fold serial dilutions of each cDNA were used for PCR with Taq polymerase (New England Biolabs), and the amplification was limited to 28 to 30 cycles. For normalization, RT-PCR with the constitutive gene *em-elp*[47] was performed.

For the cloning of gene fragments for whole-mount *in situ* hybridization, to confirm the complete coding domain sequences (CDS) of genes and for long-range PCR with genomic DNA, KOD Hot Start polymerase (Millipore) was used following the manufacturer’s instructions. In the case of *em-ago2-A*, the 5′-region of the gene is interrupted by the end of the genomic scaffold. We obtained most of the 5′-region of the CDS by taking advantage of the high similarity between the *em-ago2* genes, using a combination of a specific *em-ago2-A* primer with a primer for the 5′end of *em-ago2-B*. PCR products for sequencing and probe synthesis were cloned into pDrive (Qiagen, Hilden, Germany) or pJet1.2 (Thermo Scientific, Schwerte, Germany).

### Alkaline phosphatase histochemistry

Alkaline phosphatase histochemistry was done in cryosections and whole-mounts with nitro blue tetrazolium chloride and 5-bromo-4-chloro-3-indolyl phosphate (NBT/BCIP) as described by Cox and Singer [48].

### Immunohistochemistry and immunohistofluorescence

Immunohistochemistry and immunohistofluorescence in paraplast sections and cryosections, and whole-mount immunohistofluorescence were performed as previously described [40,49]. For anti-PHB1 and anti-H3S10-P, a step of heat induced epitope retrieval was included after re-hydration, by boiling the slides for 20 minutes in a microwave in a solution of 10 mM sodium citrate, pH 6.0 with 0.1% Triton X-100.

Primary antibodies used were Anti-PHB1 (rabbit polyclonal, Sigma-Aldrich HPA003280, 1:100 dilution), anti-phospho-histone H3 (Ser10) (rabbit polyclonal, Cell Signaling Technology, Frankfurt/Main, Germany, code 9701, 1:100 dilution), anti-FMRFamide (Immunostar, Hudson, USA, code 20091), anti-HMW-tropomyosin ([49,50], 1:500 dilution) and anti-acetylated tubulin (mouse monoclonal, clone 6-11B-1, Santa Cruz Biotechnology, Heidelberg, Germany, 1:100 dilution). In the case of anti-PHB1, we also performed a Western blot analysis with protein extracts of *E. multilocularis* metacestodes that confirmed that the antibody recognized a protein of the expected size. Secondary antibodies used were anti-mouse conjugated to FITC, anti-rabbit conjugated to FITC and anti-rabbit conjugated to peroxidase (Jackson ImmunoResearch, West Grove, PA, USA).

### Whole-mount *in situ* hybridization (WMISH)

Digoxigenin-labeled probes were synthesized by *in vitro* transcription with T7 or SP6 polymerases (New England Biolabs), using the DIG RNA labeling mix (Roche) as described by the manufacturer, from a fragment of the relevant gene cloned into pDrive or pJet1.2 (previously linearized by digestion after the gene fragment with the appropriate restriction enzyme). A list of the probes used and their lengths is described for each gene in Additional file 1. The probes were then purified with the RNeasy Mini Kit (Qiagen), checked by agarose gel electrophoresis and quantified by comparison of serial dilutions in a dot blot with the DIG-labeled Control RNA (Roche).

The WMISH protocol was adapted from the one used in the laboratory of Peter Olson (http://www.olsonlab.com/). All solutions used up to the hybridization step were RNAse free by treatment with diethyl pyrocarbonate (DEPC). The metacestode vesicles (with or without developing protoscoleces) were opened with a syringe tip and fixed in PFA-PBS overnight at 4°C. The next day, the samples were washed twice in 100% methanol, and kept at 20°C in methanol until further use. The vesicles were then transferred to 100% ethanol, rehydrated by successive steps in 75% and 50% ethanol in PBS, and washed extensively with PBS plus 0.1% Tween-20 (PBS-T). The tissue was then permeabilized with 15 μg/ml Proteinase K (Fermentas) in PBS-T for ten minutes, rinsed twice for five minutes in 0.1 M triethanolamine (TEA), pH 8, and treated twice with 0.25% v/v acetic anhydride in TEA buffer for five minutes. After washing twice for five minutes with PBS-T, the samples were re-fixed for 20 minutes in PFA-PBS at room temperature and washed extensively with PBS-T.

Samples were then transferred to pre-hybridization buffer (50% formamide, 5 X saline-sodium citrate buffer (SSC) buffer [51], 1 mg/ml *Torula* yeast RNA, 100 ug/ml heparin, 1 X Denhardt’s solution, 0.1% Tween-20, and 0.1% 3-[(3-cholamidopropyl)dimethylammonio]-1-propanesulfonate (CHAPS); all components were obtained from Sigma-Aldrich). The buffer was changed twice before pre-hybridizing for 6 to 24 hours at 60°C. Probes were then denatured by heating at 80°C for three minutes and placing directly on ice for three minutes, and added to the specimens at a concentration of 0.2 to 2 ng/ul. Hybridization was performed at 53 to 54°C (for the shorter probes for *em-muc-1* and *em-h2b* of *circa* 200 bp) or at 57 to 58°C (for other probes) for 16 to 24 hours with constant shaking.

After hybridization, the samples were washed twice with the pre-hybridization buffer for ten minutes at 57°C, three times in SSC 2X plus 0.1% Tween-20 for 20 minutes at 57°C, and three times with SSC 0.2X plus 0.1% Tween-20 at 57°C. Samples were then transferred to room temperature, washed twice with maleic acid buffer (MAB-T: 100 mM maleic acid, 150 mM NaCl, 0.1% Tween-20) and blocked for two hours at room temperature with blocking buffer (MAB-T plus 1% w/v blocking reagent for nucleic acid hybridization and detection, (Roche), and 5% v/v heat-inactivated sheep serum (Sigma-Aldrich)). Then they were incubated overnight with shaking at 4°C with anti-digoxigenin antibodies conjugated to either alkaline phosphatase or to peroxidase (Roche) in blocking buffer without sheep serum.

Finally, samples were extensively washed with MAB-T and development was performed with NBT/BCIP for alkaline phosphatase conjugated antibodies (conventional WMISH), or with fluorescein-tyramide for peroxidase antibodies, prepared and used as described by Hopman *et al*. [52] (fluorescent WMISH). Control sense probes were also used for all genes except *em-tpm-1.hmw*, and at least one control sense probe was included in all WMISH experiments, with no resulting signal (examples are included in Additional file 2).

### Fluorescence microscopy

Samples were analyzed by confocal microscopy (Leica TCS SP5; Leica Microsystems, Wetzlar, Germany) and by epifluorescence microscopy (ZeissAxio Imager.Z1 (Zeiss, Hamburg, Germany) and Keyence BZ9000 (Keyence, Neu-Isenburg, Germany)). For the quantification of EdU + and AcTub + cells, at least four random microscopic fields were captured for each whole-mount metacestode vesicle, from which the positive cells were manually counted and averaged.

### Transmission Electron Microscopy (TEM)

Protocols for TEM were performed as previously described [36].

## Results

### Cell proliferation in *E. multilocularis* larval development

**Figure 2:**
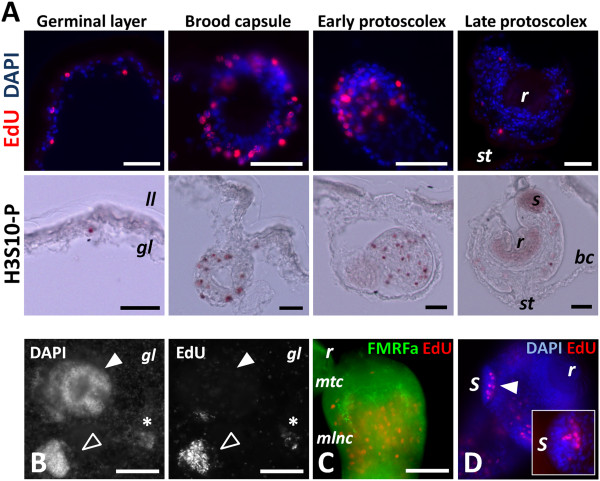
**Cell proliferation in *E. multilocularis *metacestodes. (A)** Detection of EdU incorporation and of H3S10-P in paraplast sections of different stages of development (some unspecific staining is seen in suckers and rostellum of protoscoleces for H3S10-P staining). **(B)** Whole-mount detection of EdU incorporation in a larval vesicle. The asterisk indicates an early brood capsule bud, the open arrowhead a brood capsule with a protoscolex bud, and the filled arrowhead an invaginated protoscolex. Notice also the dispersed EdU + cells in the germinative layer. **(C)** Whole-mount EdU detection (red) and FMRFamide immunofluorescence (green) during early protoscolex development. Most EdU + cells are located behind the main transverse commissure. **(D)** Whole-mount detection of EdU incorporation (red) during late protoscolex development. The arrowhead indicates the accumulation of EdU + cells at the base of the sucker. The inset shows EdU + cells in the base of a developing sucker as seen in a paraplast section. Abbreviations: bc, brood capsule; gl, germinal layer; ll, laminated layer; mlnc, main lateral nerve cord; mtc, main transverse commissure; r, rostellum or rostellum; s, sucker; st, stalk. Bars represent 30 μm except for B, 100 μm.

In order to detect proliferating cells, we incubated metacestode vesicles from *in vitro* culture with the thymidine analog 5-ethynyl-2′-deoxyuridine (EdU) [53], which is incorporated into DNA during its synthesis in the S-phase of the cell cycle, and later performed the fluorescent detection reaction on metacestode whole-mounts and Sections. A relatively long time (two hours) and high concentration of EdU (10 μM) was required for any labeling to be detected, probably because of slow equilibration between the EdU concentration in the medium and in the large amount of hydatid fluid within the vesicles. For typical labeling experiments, we used a five-hour incubation time and 50 μM EdU. EdU positive (EdU+) cells can be detected throughout the germinal layer, and are in average 5.9% of all the cells (n = 6 independent labeling experiments, with > 200 cells per experiment; range = 2.4% to 10.9%) (Figure 2A). The vast majority of the labeled cells were in interphase, but a few cases of mitotic cells with low levels of labeling were observed, suggesting that during the five-hour pulse they were labeled just at the end of the S-phase and transited through G2/mitosis (Additional file 3).

There is a strong accumulation of EdU + cells in the brood capsule buds and in the protoscolex buds (Figure 2A and B). During early development, most EdU + cells do not reach the periphery of the bud (Figure 2B). This pattern becomes more distinct as development progresses, and when the main nervous commissure becomes evident by FMRFamide immunoreactivity [40] most EdU + cells are located posterior to it (Figure 2C). In the last stages of protoscolex development, there are some EdU + cells in the posterior body, while in the scolex, EdU + cells accumulate massively at the base of the developing suckers, but not in the rest of the sucker tissue (Figure 2D). Finally, cell proliferation becomes very low when protoscolex development is complete and the scolex is invaginated (Figure 2A and B). Identical results were obtained when metacestodes that had been cultured *in vivo* in gerbils were incubated with EdU *ex vivo* immediately after removing the material from the host (Additional file 4), and similar patterns of cell proliferation have been described for protoscolex development in *E. granulosus*[37].

EdU incorporation remains very low for the first hours after protoscoleces are isolated from the metacestode material. However, when we activated protoscoleces by artificially mimicking the ingestion by the definitive host, the number of EdU + cells increased dramatically. Furthermore, prolonged *in vitro* culture of protoscoleces in the absence of activation factors also resulted in an increase of cell proliferation in many of them (Additional file 5). This indicates that in the developed protoscolex there is a large population of cells capable of proliferation, but they remain in a quiescent state or with slow cell-cycle kinetics for as long as the protoscolex remains resting within the metacestode.

As a complementary approach, we analyzed the distribution of mitotic cells by immunohistochemistry against histone H3 phosphorylated in Serine 10 (H3S10-P, [54]) after allowing the mitotic figures to accumulate by *in vitro* incubation with colchicine [24,26]. The distribution of H3S10-P + cells was identical to the distribution of EdU + cells, confirming the previous results (Figure 2A). The percentage of H3S10-P + cells in the germinative layer was low in the absence of colchicine incubation (< 0.5% of all cells), which suggests a fast transition through mitosis, as has been described in other cestodes [22,55].

### Identification of germinative cells as the only proliferating cells

**Figure 3:**
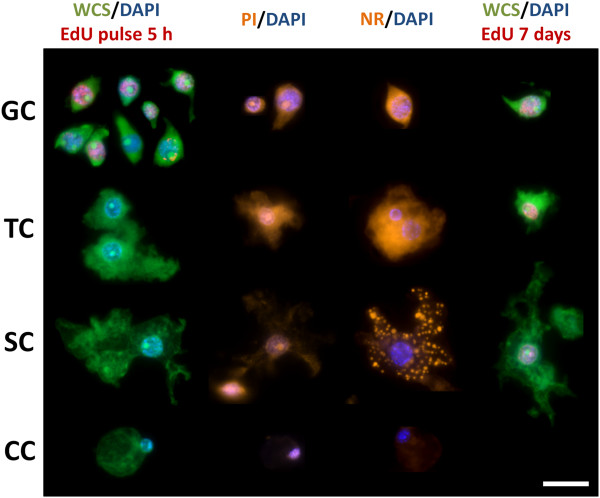
**Cell suspensions of *Echinococcus multilocularis*.** This is a montage of images of different cell types as observed in cell suspensions after different staining procedures. Cell types are indicated on the left (GC: germinative cells; TC, tegumental cells; SC, glycogen/lipid storage cells; CC, calcareous corpuscle cells) and staining procedures are indicated on top (NR, Nile red, shown in orange; PI, propidium iodide, shown in orange; WCS, whole cell staining, shown in green), including the detection of EdU (shown in red) after two different labeling treatments, 50 μM for five hours and 1 to 10 μM for seven days (see the main text for details). The bar represents 10 μm.

Because of the small size of *Echinococcus* cells and the loose organization of the germinative layer, it is very difficult to identify cell types *in situ* by morphology except by means of electron microscopy [28,56]. In order to identify the EdU + cells, we performed a tissue maceration procedure that results in a suspension of cells that retain their original morphology [44,57-59]. We then stained these suspensions with 4′,6-diamidino-2-phenylindole (DAPI, that specifically stains DNA) combined with propidium iodide (PI) or the Thermo Scientific Cellomics™ Whole Cell Stain (WCS) that stain all nucleic acids and are therefore analogous to the traditional Pyronin Y staining for basophilic, RNA-rich cells [26]. In parallel, we performed staining of cell suspensions for lipids using Nile red (NR) combined with DAPI (Figure 3).

With this method, we consistently identified the germinative cells as small (5 to 12 μm across the longest axis), pear-shaped to fusiform cells that are strongly stained with PI and WCS, and that can sometimes have thin cytoplasmic extensions protruding from the poles. The nucleus is round and very large, with one to three very prominent nucleoli and with finely granular chromatin, giving a very bright staining with DAPI. Cytoplasmic lipid droplets were rare.

The germinative cells were the only cells that incorporated EdU after two to six hours of incubation *in vitro* (n = 5 independent labeling experiments): after five hours of labeling, an average of 24% ± 6.7% (standard deviation) of the germinative cells were EdU +. Germinative cells were also the only cells observed in mitosis. Size differences were observed between these cells, and smaller germinative cells were less likely to incorporate EdU (Additional file 6), suggesting that cell size may be related in part to different cell-cycle phases. In small metacestode vesicles, the germinative cells were on average 21% of all the cells. We observed that in larger vesicles, the apparent abundance of the germinative cells was higher, up to around 50% of all cells. However, in these vesicles the tissue maceration was incomplete, and we believe that the germinative cells were overrepresented in the cell suspensions. Indeed, taking into account that in whole-mounts of these vesicles an average of 5.9% of all cells were EdU +, and that 24% of all germinative cells are EdU + in the cell suspensions, by assuming that all EdU + are germinative cells (see above) one can approximately estimate the fraction of germinative cells as 25% of all cells. In activated protoscoleces, although the tissue maceration was incomplete as well, we also observed germinative cells as the only EdU + cells after a five hour pulse (Additional file 7).

All morphologically differentiated cells were consistently EdU −, indicating that they are generated by differentiation of the proliferating germinative cells. Among the differentiated cells, we could recognize several types by comparison with ultrastructural descriptions from *E. multilocularis* metacestodes and classic histological studies in other cestodes [23,28,39,58] (Figure 3). These included: 1) the tegumental cells, with abundant cytoplasm strongly stained with PI/WCS, uniformly stained by NR, and with an irregularly shaped border. The nucleus can be slightly irregularly shaped and shows chromatin clumps in the periphery; 2) the glycogen/lipid storage cells. These cells have large and smooth cytoplasmic lobes, show very low staining with PI/WCS, and have lipid droplets as seen by NR staining; 3) the calcareous corpuscle forming cells, with a small and eccentric nucleus and a large round cytoplasm that shows little staining for PI, WCS or NR; 4) several small cell types with a small, heterochromatin-rich nucleus. It is likely that muscle cells and nerve cells are found in this category after losing their cytoplasmic extensions during the maceration procedure.

In order to confirm that the differentiated cell types are generated from the pool of proliferating germinative cells, we performed EdU pulse-chase assays, in which we incubated the vesicles for two hours with 50 μM to 100 μM EdU, followed by washing and incubation in EdU-free medium for up to seven days. Unfortunately, we observed that the EdU signal was stronger after a three-day chase period than directly after the pulse (data not shown), indicating that EdU remains in the hydatid fluid after washing. As a complementary approach, we performed continuous EdU labeling experiments with 1 μM to 10 μM EdU for up to 14 days. In this setting, we observed that the higher concentrations (10 μM) showed some toxicity in this setup, whereas lower concentrations (0.5 μM) did not yield cells with enough labeling for detection. In both pulse-chase and continuous labeling experiments, we observed EdU + tegumental and glycogen/lipid storing cells after seven days (Figure 3 and data not shown), suggesting differentiation of germinative cells into these cell types. In summary, we identified the germinative cells as the only proliferating cell population, and the evidence suggests that differentiated cell types are generated from the germinative cells.

### Gene expression patterns in the germinative cells

To identify genes that are specifically expressed in the germinative cells, we analyzed the expression of several candidate genes among planarian neoblast markers by whole-mount *in situ* hybridization (WMISH).

### em-h2b

As a possible general marker of all proliferating germinative cells, we analyzed the expression of histone H2B homologs, since canonical histones are synthesized in a cell-cycle dependent manner: histone transcripts only accumulate during the S-phase, when new histones are needed to accompany the synthesis of DNA [60]. Furthermore, H2B genes have been found to be specifically expressed in proliferating planarian neoblasts and in the neoblast-like cells of the trematode *Schistosoma mansoni*[61,62].

**Figure 4:**
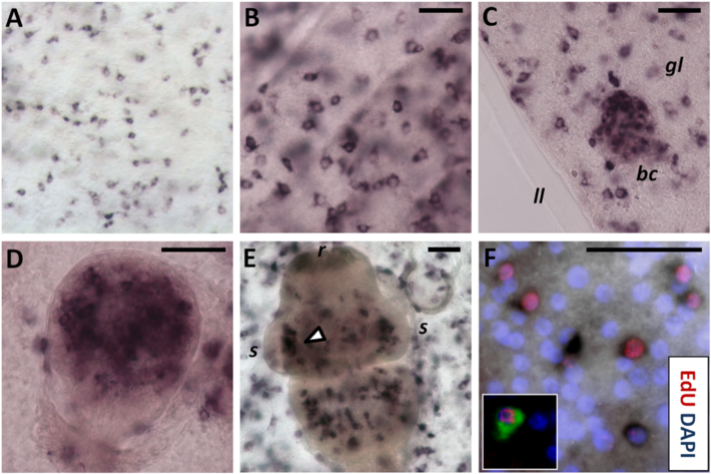
**WMISH detection of *em-h2b*. (A)** General view of the germinal layer. **(B)** Detail of the germinal layer; showing the germinative cell morphology of the positive cells. **(C)** Early brood capsule bud. **(D)** Protoscolex bud. **(E)** Late protoscolex development. The arrowhead indicates the accumulation of positive cells at the base of the sucker. **(F)** Co-localization of *em-h2b* (dark precipitate) and EdU detection (red) after a 50 μM, five hour pulse. The inset shows an example in which the *em-h2b* signal was inverted and pseudo-colored in green to facilitate the visualization of the co-labeling. Abbreviations are as in Figure 2. Bars represent 25 μm.

Several canonical H2B genes are present in the *E. multilocularis* genome. Most of them are almost identical to each other (> 95% nucleotide identity), which we denominate the *em-h2b-1* group. Another gene, *em-h2b-2*, also shows high amino acid identity (97%) but lower nucleotide identity (85%) to *em-h2b-1*. Using probes for *em*-*h2b-1* and *em*-*h2b-2* gave identical results, which were indistinguishable from the EdU labeling pattern in the germinal layer and throughout the development of brood capsules and protoscoleces (Figure 4). This is particularly striking during late protoscolex development, where a massive accumulation of *em-h2b +* cells is found at the base of the suckers but no expression is seen in the remaining sucker tissue (Figure 4E).

The WMISH signal labeled the cytoplasm of cells with the typical germinative cell morphology (Figure 4B). Furthermore, when combining WMISH with EdU detection after a five hour pulse, 78% of all *em-h2b +* cells were also EdU + (n = 197 *h2b* + cells), and conversely, 87% of all EdU + cells were also *em-h2b +* (n = 176 EdU + cells) (Figure 4F). Because only germinative cells proliferate (see above), *em-h2b* is therefore a *bona fide* marker of S-phase germinative cells, but would not detect resting or G1 and G2/M germinative cells. The smaller proportion of EdU − *h2b* + cells had probably already entered S-phase but were fixed before enough EdU was incorporated for detection, while the EdU + *h2b −* cells were probably fixed after they had already incorporated EdU but exited the S-phase during the incubation time.

### *em-nos-1* and *em-nos-2*

**Figure 5:**
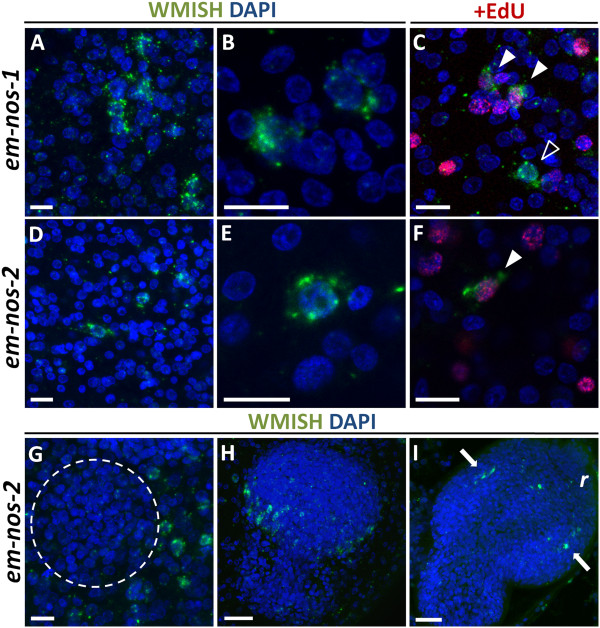
**WMISH detection of *em-nos-1 *(A-C) and *em-nos-2 *(D-I).** A and D, general view and B and E, detail of the positive cells in the germinative layer. C and F, co-localization in the germinal layer of *em-nos-1* and *em-nos-2* (green) with EdU incorporation (red) after a five hour 50 μM pulse. Double positive cells are indicated with a filled arrowhead, whereas cells expressing a *nanos* gene but EdU − are indicated with an open arrowhead. G, Expression of *em-nos-2* in cells surrounding a brood capsule bud (dashed circle). H, Protoscolex bud. I, Later protoscolex development. Arrows indicate *em-nos-2*+ cells in the position of the developing lateral ganglia. Abbreviations are as in Figure 2. Bars represent 10 μm except for H, 20 μm.

We then turned to possible post-transcriptional regulators of the germinative cells. *nanos* genes are molecular markers of the germ line in many classical models, but are also expressed in multipotent stem cells in various basal metazoan lineages [11,19,63,64]. Two *nanos* genes are present in *E. multilocularis* (*em-nos-1* and *em-nos-2*). Both genes were expressed in few cells with a patchy distribution in the germinal layer (Figure 5A and D), and with the morphology of large germinative cells (< 1.6% of all cells for both *em-nos-1* and *em-nos-2*, n = 4,632 cells and n = 7,475 cells, respectively; Figure 5B and E). Furthermore, *em-nos-1* and *em-nos-2* cells can incorporate EdU (19% of *em-nos1*+ cells are EdU + after a five hour pulse, n = 96), although the vast majority of EdU + cells do not express either gene (< 5% of all EdU + cells express either *nanos* gene, Figure 5C and F). Altogether, these data show that a small subpopulation of the germinative cells in the germinal layer express *em-nos-1* and *em-nos-2*, although it is not clear if both genes are co-expressed in the same cells.

During brood capsule and protoscolex development, *em-nos-1* expression was not detected. Expression of *em-nos-2* was sometimes seen around brood capsule buds, and later during early protoscolex development as a small population of cells at the base of the protoscolex bud (Figure 5G and H). Finally, *em-nos-2* is expressed in a few cells associated with the developing nervous system, in the region of the developing lateral ganglia and the main commissure (Figure 5I). These results show that most proliferating cells do not express *nanos* genes in the developing protoscolex, and suggest a role for *em-nos-2* during nervous system development.

### em-ago2

Although *piwi* genes are not present in *E. multilocularis*, there are other argonaute proteins encoded by the genome: an ortholog of human Ago-1-4 proteins which is likely involved in RNA interference (EmAgo1 [65]) and three copies of an Argonaute gene family that is specific for cestodes and trematodes [38], which we dubbed *em-ago2-A* to *em-ago2-C*. We further identified a pseudogene, *em-ago2-*Ψ (Additional file 8). These copies have resulted from a recent duplication that occurred after the divergence of *Hymenolepis* and -*Echinococcus* + *Taenia*- (see the phylogenetic analyses in [38]), with 88 to 99% nucleotide sequence identity between copies (depending of the copies and the specific regions compared). Moreover, they are organized as two couples of tandemly arranged copies in close proximity to a copy of a *Sec61* homolog (Additional file 8). This conservation of synteny suggests that one first duplication occurred which resulted in two adjacent copies of an original *em-ago2* gene located next to a *Sec61* gene, followed by the duplication of the whole region. Long-range PCR with genomic DNA confirmed the organization of these genomic regions (Additional file 8), while sequencing of smaller PCR fragments confirmed the existence of all four copies, demonstrating that they are not an artifact of genome assembly. By RT-PCR only *em-ago2-A*, *em*-*ago2-B* and *em-ago2-*Ψ mRNA could be detected, whereas *em-ago2-C* was absent or barely detected in all of the larval stages and in primary cell cultures.

**Figure 6:**
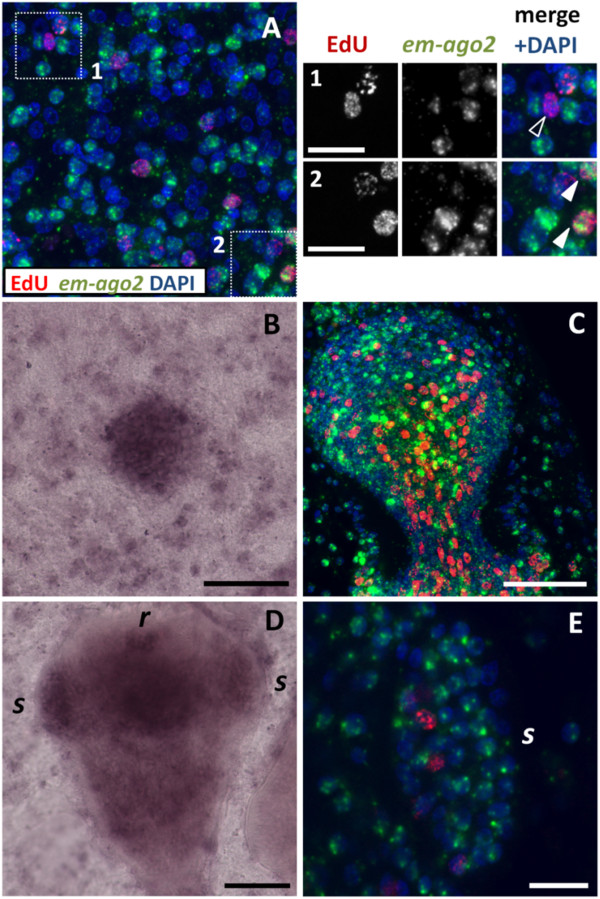
**WMISH detection of *em-ago2*. (A)** General view of the germinal layer. The insets show details of EdU + *em-ago2* cells (open arrowheads) and EdU + *em-ago2*+ cells (filled arrowheads) after a five hour, 50 μM EdU pulse. **(B)** Early brood capsule bud. **(C)** Protoscolex bud; colors are coded as in Figure 5A. **(D)** Late protoscolex development. **(E)** Detail of a sucker from a protoscolex in late development, showing that *em-ago2* expression is not restricted to the base; colors are coded as in Figure 5A. Abbreviations are as in Figure 2. Bars represent 10 μm in A1 and A2, and 40 μm in all other panels.

We performed WMISH using two different probes for *em-ago2-A*. These probes would probably cross-react with all other *em-ago2* copies, and we refer to the expression pattern of all these genes by the collective name *em-ago2. em-ago2* expression was similar to the pattern of EdU incorporation in the germinal layer, and cells strongly expressing *em-ago2* accumulate in the brood capsules and protoscolex buds (Figure 6A to C). The distribution of the *em-ago2* signal within cells is very distinct since it is only observed close to or within the nucleus (Figure 6A). Strongly positive *em-ago2*+ cells account for approximately 30% of all cells in the germinal layer, but notably, some expression of *em-ago2* was observed in more than 50% of all cells, indicating that it is not exclusive to the germinative cell population.

There is no clear-cut correlation between the level of *em-ago2* expression and proliferation, since approximately 50% of EdU + cells show low or no expression of *em-ago2* (Figure 6A). Conversely, during early protoscolex development, it is clear that although *em-ago2* expression is present in most cells, the cells with the strongest *em-ago2* signal are almost invariably EdU − (Figure 6C). During late protoscolex development, some *em-ago2* signal is observed in most cells (Figure 6D and E), and is not restricted to the base in the suckers (where cell proliferation occurs). It is therefore clear that the expression of *em-ago2* is not restricted to proliferating cells. Moreover, the expression of *em-nos-1*, *em-nos-2* and *em-ago2* point to extensive heterogeneity at the molecular level among the proliferating germinative cells.

### *em-hdac1* and *em-phb1*

The histone deacetylase HDAC1 is one of many chromatin modifying proteins that are specifically expressed in planarian neoblasts [15,66]. Neoblast specific expression has also been shown for the mRNA of homologs of prohibitin-1 and prohibitin-2 [15,16]. In mammalian cells, prohibitins form complexes in the inner mitochondrial membrane with unclear biochemical function, and have been linked to mitochondrial biogenesis and cell proliferation [67].

We found single-copy orthologs of HDAC1 (*em-hdac1*) and prohibitin-1 (*em-phb1*) in the *E. multilocularis* genome, and both genes showed widespread expression in the germinal layer and during protoscolex development (Additional files 9 and 10). We also determined the distribution of the Em-PHB1 protein by immunohistochemistry, and compared this to the distribution in planarian tissues using a commercial antibody that recognizes a conserved region in all PHB1 proteins. In planarians, although low levels of PHB1 are seen in post-mitotic tissues such as the pharynx, the highest signal is observed in neoblast-like cells in the mesenchyma (Additional file 11). In contrast, *E. multilocularis* Em-PHB1 is observed throughout the germinal layer, brood capsules and developing protoscoleces (Additional file 12). In summary, neither gene has a germinative cell specific expression in *E. multilocularis* metacestodes.

### Identification of molecular markers for differentiated cell types

The identification of differentiated cell types in the germinal layer is difficult, and unlike the situation in adult cestodes, trematodes and planarians, the lack of spatial segregation of any post-mitotic cell types makes it impossible to trace the differentiation of germinative cells *in situ.* Therefore, we set out to find molecular markers of differentiated cell types in *Echinococcus*.

### *em-muc-1* and *em-alp-2* as tegumental cell markers

Since the laminated layer is synthesized by the tegumental syncitium, genes coding for laminated layer components should be expressed by the tegumental cells [68]. We analyzed the expression of *em-muc-1*, a member of an *Echinococcus*-specific, highly expressed apomucin gene family that has been proposed to be a main component of the laminated layer [38,68,69]. Because all members are very similar (with regions with over 90% identity at the nucleotide level) it is likely that the *em-muc-1* probe recognizes most genes of this gene family.

**Figure 7:**
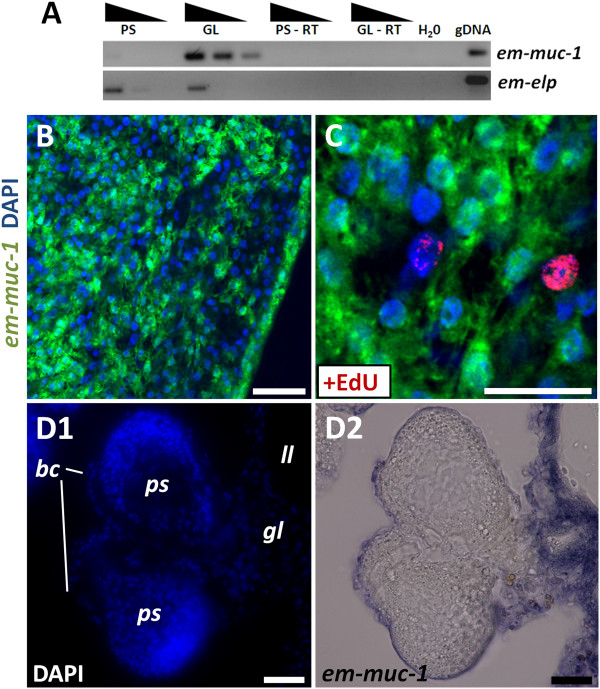
**Expression of *em-muc-1*. (A)** Semi-quantitative RT-PCR with serial ten-fold dilutions of cDNA from protoscoleces (PS) and germinal layer (GL). Controls without reverse transcriptase (RT) and without template (H_2_0) are included. **(B-D)** WMISH of *em-muc-1*. (B) General view of the germinal layer. (C) Double detection of WMISH (green) and EdU incorporation after a five hour, 50 μM EdU pulse (red); note the lack of EdU labeling among the *em-muc-1*+ cells. (D1 and D2) Section of metacestode processed for WMISH, showing the lack of expression in the developing protoscoleces, strong expression in the germinal layer, and expression in the brood capsule wall. Abbreviations are as in Figure 2. Bars represent 20 μm except for B, 10 μm.

*em-muc-1* is strongly expressed in the germinal layer but not in protoscoleces, as is expected for a component of the laminated layer (Figure 7A). *em-muc-1* was expressed in cells with abundant cytoplasm that fuse or interdigitate with each other, and which constitute 27 to 37% of all cells in the germinative layer (two independent WMISH experiments; n = 3,440 and 780 cells, respectively; Figure 7B). In stark contrast, no expression is observed in early brood capsule buds or in developing protoscoleces. To our surprise, however, we could detect low levels of *em-muc-1* in late brood capsules, which suggests that the glycocalix of the brood capsule may also contain the product of *em-muc-1*, although the laminated layer does not line the brood capsule cavity [68] (Figure 7D). By combining WMISH with EdU detection, we observed the absence of EdU + cells among the *em-muc-1*+ cell population (n = 1,454 *em-muc-1*+ cells from two independent WMISH experiments; Figure 7C). We conclude that *em-muc-1* is a robust marker for tegumental cells in the germinal layer, and confirm that the tegumental cell population does not proliferate.

**Figure 8:**
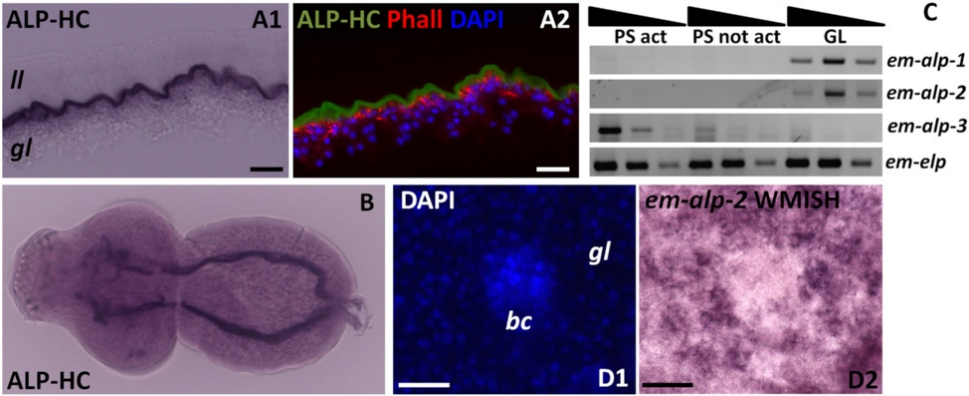
**Alkaline phosphatase activity and gene expression. (A1)** Alkaline phosphatase histochemistry in the germinal layer, showing strong activity in the syncitial tegument. **(A2)** The signal in A1 was inverted and pseudo-colored in green, and combined with DAPI (blue) and phallodin (red) staining to show the distribution of the nuclei and muscle fibers, respectively. **(B)** Alkaline phosphatase histochemistry in an activated protoscolex, showing activity in the excretory system. **(C)** Semi-quantitative RT-PCR with serial ten-fold dilutions of cDNA from activated protoscoleces (PS act), non-activated protoscoleces (PS not act) and germinal layer (GL). The experiment was repeated three times with similar results. **(D)** WMISH of *em-alp-2,* showing strong expression in the germinal layer but not in the brood capsule bud. Abbreviations are as in Figure 2. Bars represent 20 μm.

While searching for possible histochemical markers, we observed that alkaline phosphatase activity in the metacestode is very high (strong reaction in less than five minutes) and restricted to the distal syncitial tegument of the germinal layer (Figure 8A; see also [70]), but is not found in brood capsules (Additional file 13). This indicated that one or more alkaline phosphatase genes must be expressed in the tegumental cells. In protoscoleces, alkaline phosphatase was detected only after several hours, and only in the excretory system (Figure 8B), similar to what has been described in the developing adult [71]. This activity increased after protoscolex activation (Additional file 13). Four genes encoding alkaline phosphatases (*em-alp-1* to *em-alp-4*) were found in the *E. multilocularis* genome. By RT-PCR, *em-alp-1* and *em-alp-2* were found to be specifically expressed in the germinal layer, while *em-alp-3* was only detected in protoscoleces, with a strong up-regulation after protoscolex activation (Figure 8C). *em-alp-4* has substitutions of conserved catalytic amino acid residues, and no expression was detected by RT-PCR in the germinal layer or in protoscoleces, suggesting that it is a pseudogene, although expression was observed in high throughput RNA sequencing data of adult worms [38]. Altogether, the data suggested that *em-alp-1* and *em-alp-2* were expressed in the tegumental cells of the germinal layer, while *em-alp-3* was expressed in the protoscolex excretory system, and is to the best of our knowledge the first gene shown to be up-regulated after protoscolex activation. We therefore analyzed the expression pattern of *em-alp-2* by WMISH, and found it to be identical to that of *em-muc-1* in the germinal layer, with no expression in the brood capsule buds or in the developing protoscoleces (Figure 8D). In conclusion, *em-alp-2* is another marker for the tegumental cells in the germinal layer.

### Acetylated tubulin α as a marker of nerve cells

We have recently shown that a net of nerve cells in the germinal layer can be detected by immunohistofluorescence against acetylated tubulin α (AcTub + cells, [40]). Here, we show that the nerve cells do not proliferate, since they are EdU − after a five hour, 50 μM EdU pulse (n = 874 AcTub + cells from three independent experiments; Additional file 14). Similarly, in activated protoscoleces all AcTub + cells (including in this case both nerve cells and flame cells [40]) were EdU − (data not shown).

We performed a quantitative analysis of the formation of new AcTub + cells by determining the percentage of EdU + AcTub + cells during continuous EdU labeling. No double labeled cells were observed after seven days, but the percentage increased to 13.3% after 14 days (Additional file 14). Because no EdU incorporation was observed in nerve cells even after seven days of continuous exposure, this strongly indicates that all EdU + nerve cells must originate from the differentiation of proliferating germinative cells, which would require more than seven days after exiting the cell cycle to become AcTub+. Higher concentrations of EdU (10 μM) apparently had a toxic effect, and only 2.8% of the AcTub + cells were EdU + after 14 days (Additional file 14).

### *em-tpm-1.hmw* as a marker for muscle cells during protoscolex development

Using a specific antibody that recognizes the high molecular weight (HMW) isoforms of two tropomyosin genes from cestodes (*tpm-1* and *tpm-2*), HMW-tropomyosins have been shown to be present exclusively in the muscle fibers in the cestode *Mesocestoides corti*, and are strongly expressed in the suckers of *E. granulosus* protoscoleces [49,50]. Using this antibody, we confirmed that HMW tropomyosin isoforms can be found in the muscle fibers in the germinal layer, accumulating in the interior of brood capsules and in the muscle layers during protoscolex development in *E. multilocularis* (Additional file 15), in perfect correlation to the description of muscle fibers as determined by phalloidin labeling [40].

**Figure 9:**
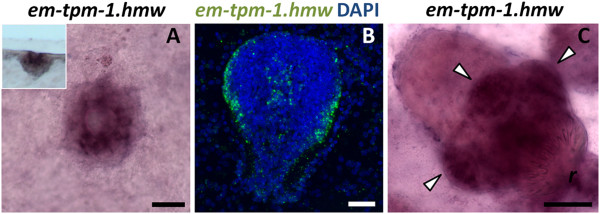
**WMISH detection of *em-tpm-1.hmw*. (A)** Early brood capsule bud as seen from above. The inset shows a lateral view. **(B)** Protoscolex bud. **(C)** Developed protoscolex. The arrowheads point to the suckers. Abbreviations are as in Figure 2. Bars represent 20 μm (A, B) or 40 μm (C).

Because in cestodes the nucleus of muscle cells is located in a non-contractile cell body, connected by thin cytoplasmic bridges to the contractile myofibers [72,73], it is not possible to identify the cell bodies by immunodetection of HMW-tropomyosins. Instead, we analyzed the expression of the HMW isoform of *em-tpm-1* by WMISH using a specific probe. Surprisingly, no expression was observed in the germinal layer, suggesting that muscle cells in this tissue express other tropomyosin isoforms (that is from *em-tpm-2*). Instead, *em-tpm-1.hmw* was detected in individual cells in the center of invaginating brood capsules (Figure 9A), in close proximity to the location of muscle fibers [40]. During early protoscolex development, when muscle development is already underway [40], *em-tpm-1.hmw* is expressed in two symmetrical bands of superficial cells and one internal medial band (Figure 9B). Because of their distribution, these are likely to be the subtegumental circular muscle cells and the inner longitudinal muscle cells. Finally, strong expression was observed in the muscular suckers and rostellum and in individual cells in the body of the developed protoscolex (Figure 9C). In summary, *em-tpm-1.hmw* can be used as a molecular marker for the development of muscle cells during brood capsule and protoscolex development, but not in the germinal layer.

### Partial depletion of germinative cells by irradiation and hydroxyurea treatments

**Figure 10:**
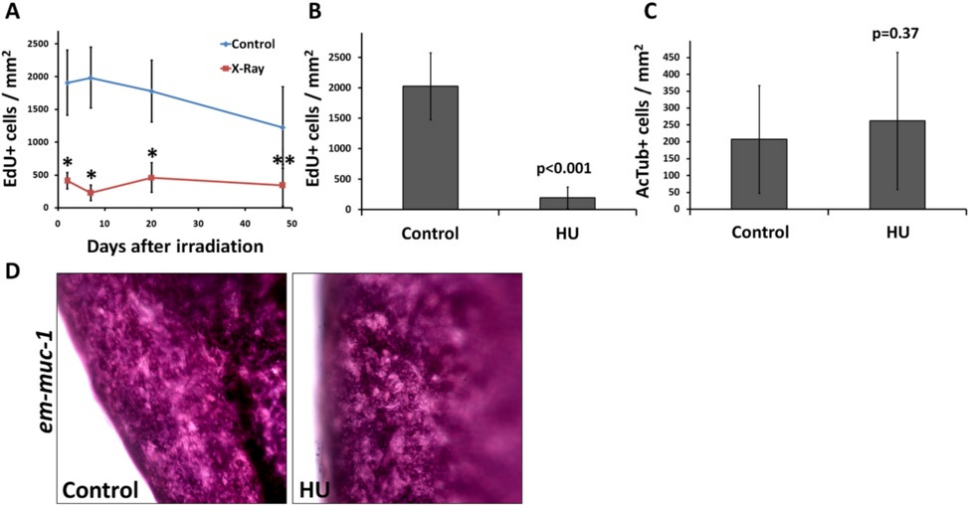
**Effect of X-ray irradiation and hydroxyurea (HU) treatment. (A)** Number of EdU + cells per area at different times after X-ray irradiation (150 Gy) and in unirradiated controls, after a five hour 50 μm EdU pulse (average and standard deviations of 7 to 14 vesicles per time point). **(B)** Number of EdU + cells per area in vesicles treated with HU (40 mM for seven days) and in non-treated controls (all vesicles were allowed to recover for 24 hours in HU-free medium, followed by a five hour 50 μm EdU pulse; average and standard deviation of seven independent experiments). **(C)** Number of AcTub + cells per area in vesicles treated with HU (40 mM for seven days) and in non-treated controls (average and standard deviation of nine to eleven vesicles pooled from three independent experiments). **(D)** WMISH of *em-muc-1* in a vesicle treated with HU (40 mM for seven days) and in a non-treated control. *P < 0.001,**P < 0.01. The Mann–Whitney *U*-test was used for A-C.

Total and partial elimination of neoblasts by irradiation has been a powerful tool for studying neoblast gene expression and physiology in many flatworms [7,13,15,16,62,74,75]. In *E. multilocularis*, comparable doses of ionizing radiation (50 to 100 Gy) were found to only retard growth of metacestodes, and did not eliminate the germinative cells [76]. We performed similar experiments with a single X-ray irradiation dose of 150 Gy on metacestode vesicles (lacking brood capsules and protoscoleces). At 48 hours post-irradiation, we observed that the number of cells incorporating EdU per area of germinative layer decreased in average to 22% of unirradiated controls, showing that very high dosages of X-ray irradiation decrease but are unable to eliminate all proliferating cells (Figure 10A). Interestingly, we observed no significant increase in the number of EdU incorporating cells for up to 48 days post-irradiation, and the number of EdU + cells per area was still on average only 28% that of unirradiated controls (Figure 10A). Despite this long-term decrease in cell proliferation, no difference in survival was observed between irradiated and unirradiated metacestodes after 48 days (82% versus 86%, *P* = 0.49, Chi-square test).

As an alternative approach, we used hydroxyurea (HU), an inhibitor of ribonucleotide reductase (RRM) that is specifically toxic to cells undergoing DNA synthesis during cell proliferation [77-79], and which has also been used in other invertebrates to eliminate stem cells [75,80,81]. The mechanism of toxicity is based on the depletion of deoxyribonucleotide triphosphates (dNTPs) that results from RRM inhibition, provoking the cessation of DNA replication and thus leading to stalled replication forks and eventually to chromosomal DNA damage [82]. In order to confirm a similar effect of HU on *E. multilocularis* cells, we incubated primary cell cultures with different HU concentrations. Indeed, incorporation of the thymidine analog 5-bromo-2′-deoxyuXridine (BrdU) was reduced by approximately 50% and 90% in the presence of 10 mM and 40 mM HU, respectively. Furthermore, the regeneration of metacestode vesicles from primary cells was strongly decreased by 10 mM HU and abolished by 40 mM HU (Additional file 16).

We therefore incubated metacestode vesicles (lacking brood capsules and protoscoleces) with 40 mM HU for seven days, and allowed them to recover in HU-free media for 24 hours. This resulted on average in a 90% reduction in the number of cells incorporating EdU per area of germinative layer (Figure 10B). Furthermore, the experimental results could be divided into two groups: Group 1, from experiments performed on larger and older vesicles, resulted in a decrease of only 66% to 93% in the number of EdU + cells (similar to the results observed after X-ray irradiation), whereas Group 2, performed in smaller and younger vesicles, resulted in a greater decrease of 97.7% to 99.8% of EdU + cells with respect to the non-treated controls.

We analyzed in detail the effect of HU on the metacestode vesicles. Quantification of germinative cells in tissue macerates showed that the loss of EdU + cells was paralleled by a decrease in germinative cells, from 20 to 22% to 3 to 5% of all cells (two independent experiments, *P* ≤ 0.001 for both, Chi-square test). However, differentiated cells did not seem to be affected by the HU treatment: nerve cell numbers per area of germinative layer were not significantly reduced, as determined by AcTub immunohistofluorescence (Figure 10C) and the tegumental cell marker *em-muc-1* showed a qualitatively similar expression pattern in both conditions (Figure 10D). We conclude that HU treatment for seven days specifically depletes the germinative cell population, with little effect on the number of differentiated cells.

**Figure 11:**
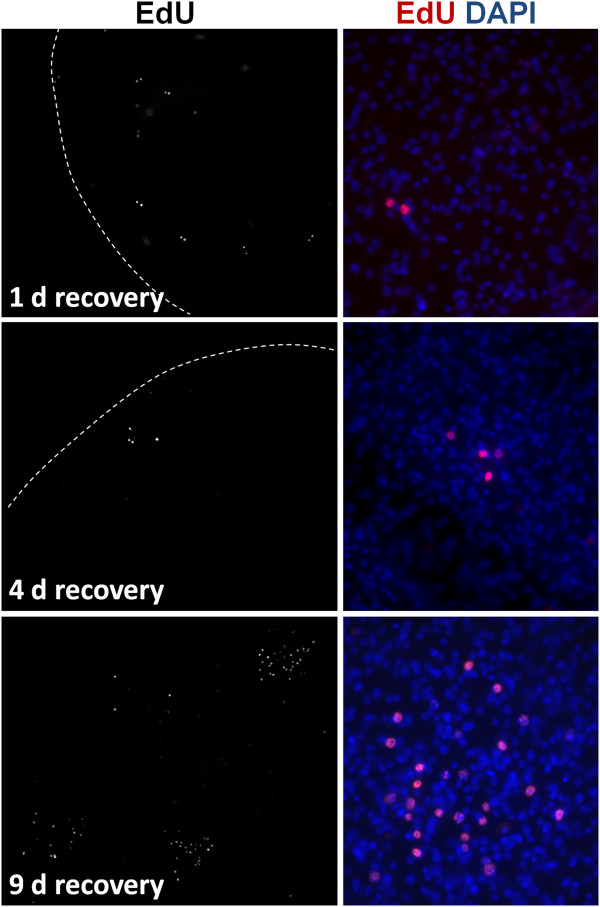
**Growing patches of EdU + cells during recovery from hydroxyurea (HU) treatment.** Vesicles were treated for seven days with 40 mM HU, transferred to HU-free medium and samples were taken for EdU labeling (five hours with 50 μM EdU) and detection at the indicated times of recovery.

Prolonged times of recovery of vesicles from Group 1 in HU-free media did not result in a significant increase of EdU + cells after up to 22 days, similar to the results obtained after X-ray irradiation. However, in Group 2, we could observe highly localized, time-dependent increments in the number of EdU + cells, strongly suggestive of clonal growth from surviving proliferating cells (Figure 11). In most experiments, only isolated EdU + cells could be found after one day of recovery. As recovery time increased, patches of two EdU + cells (after one to four days of recovery), three to four EdU + cells (after four to nine days of recovery) and of more than thirty EdU + cells (after nine days of recovery) could be found in some of the metacestode vesicles. This strongly indicates the existence of cells in the metacestode (presumably germinative cells) that can respond to the substantial depletion of proliferating cells by undergoing self-renewing divisions into proliferation-competent cells.

### Germinative cells proliferate and are enriched in primary cell preparations

**Figure 12:**
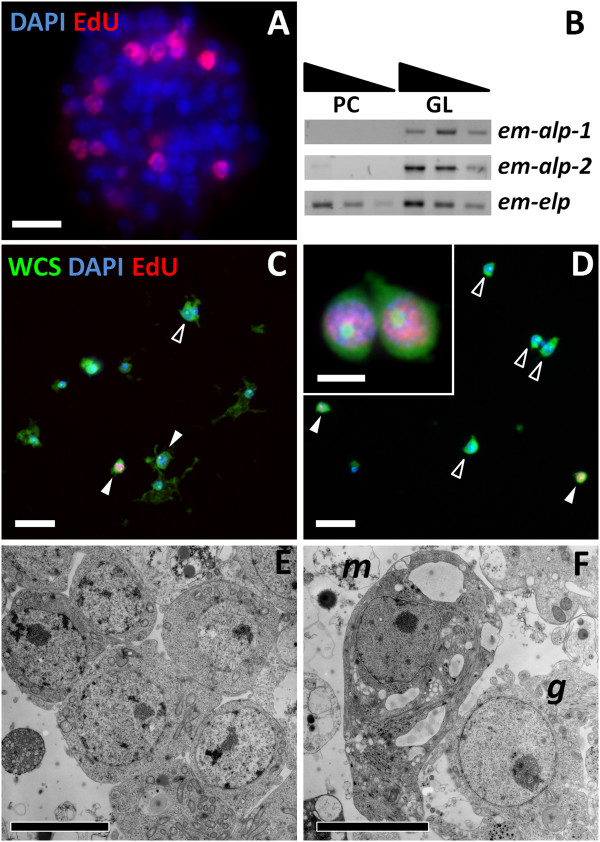
**Characterization of early primary cell preparations. (A)** EdU incorporation after a 50 μm five hour pulse of primary cell aggregates (two days-old). **(B)** Semi-quantitative RT-PCR of *em-alp-1* and *em-alp-2* genes with serial ten-fold dilutions of cDNA from primary cells (PC) and germinal layer (GL). The experiment was repeated three times with similar results. **(C** and **D)** Representative microscopy fields of cell suspensions obtained from the germinal layer (C) and from primary cells (D). EdU + and EdU − germinative cells are indicated by filled and open arrowheads, respectively. The inset in D shows a close-up of two EdU + germinative cells in the primary cell preparations. **(E** and **F)** Transmission electron microscopy (TEM) of primary cell aggregates (two days-old). **(E)** Accumulation of germinative cells. **(F)** A muscle cell (m), containing myofibers and extensive smooth endoplasmic reticulum, and a germinative cell (g) in the periphery of an aggregate. Notice also cell debris surrounding the cells. Bars represent 4 μm (A, E, F and inset in D) and 20 μm (C and D).

In the previously described primary cell regeneration system [36], primary cells obtained from *E. multilocularis* metacestodes initially form small aggregates that grow and fuse to each other. Within these aggregates fluid-filled cavities are formed, and eventually new metacestode vesicles are generated by a still incompletely understood process. We analyzed cell proliferation within the early aggregates (after two days of culture) by EdU labeling. We observed extensive cell proliferation in a layer within the aggregates, but the innermost cells did not incorporate EdU, suggesting that they have exited the cell-cycle, which may be related to the initial formation of internal cavities (typically observed after four days of culture) (Figure 12A). In order to morphologically identify the proliferating cells during regeneration, we prepared cell suspensions by the tissue maceration procedure, and based the identification of cell types on the previously described morphological criteria. As found in metacestodes, germinative cells were the only cell type labeled by EdU in primary cell preparations (Figure 12C and D). Furthermore, germinative cells were enriched in two day-old aggregates, comprising 62% to 83% of all cells, as compared to 32% to 55% in the metacestodes that were used to generate the primary cell preparations (three independent primary cell preparations, *P* < 0.02 for all preparations, Chi-square test; furthermore, note that the percentage of germinative cells is likely overestimated for the metacestodes since large vesicles were used). Among the differentiated cells, tegumental and glycogen/lipid storage cells were conspicuously few, and an increase in cells with degenerating morphology was noted. Further confirmation of the substantial depletion of tegumental cells was obtained by analyzing the expression of the tegumental cell markers *em-muc-1*, *em-alp-2* and of *em-alp-1*. Preliminary high throughput RNA sequencing data indicated low expression levels for all three genes in primary cells (2%, 7% and 0% of the levels found in the germinal layer, respectively), that was confirmed by semi-quantitative RT-PCR for *em-alp-1 and em-alp-2* (Figure 12B).

To confirm the enrichment of germinative cells and to determine which other cell types are present in early primary cell aggregates, we performed transmission electron microscopical studies of two day-old primary cell aggregates. Abundant germinative cells could be found in the aggregates, surrounded by an external layer of cells showing signs of degeneration (necrosis) (Figure 12E and Additional file 17). Also, many muscle fibers and some muscle cells could be identified (Figure 12F). In cryosections of primary cells after three days of culture, muscle fibers were also found by phalloidin staining, and nerve cells were identified by AcTub immunohistofluorescence (Additional file 18). In summary, early primary cell preparations are enriched in proliferating germinative cells, but other cell types such as muscle cells and nerve cells are also present.

## Discussion

### Germinative cells are the only proliferating cells in *Echinococcus multilocularis* larvae

The germinative cells in *E. multilocularis* constitute a morphologically homogeneous population, similar to descriptions in other cestode species and life stages [21-24,27], and generally similar to the neoblasts in free living flatworms and the neoblast-like cells of the trematode *Schistosoma mansoni*[5,62]. The main differences observed within the germinative cells in *E. multilocularis* were related to the number of nucleoli, size, and the presence or absence of thin cytoplasmic projections. All of these differences could be related in part to normal changes during the cell cycle and cell migration [83,84], although other authors have sub-divided the germinative cells into different types based on size and histological details [21,27]. Germinative cells were identified as the only proliferating cell type during metacestode growth and regeneration, indicating that all new cells must originate from this population. However, the existence of de-differentiation and trans-differentiation processes in differentiated cells cannot completely be ruled out at this point. Unambiguous lineage tracing will require analyses mediated by stable genetic markers, and current efforts in our laboratory are being made towards transgenic modification of *Echinococcus* germinative cells. Furthermore, the markers for differentiated cell types developed in this work will be an important tool for tracing the differentiation pathways of germinative cells.

Unfortunately, so far we have been unable to find a universal molecular marker for the germinative cells. However, H3S10-P and *em-h2B* are useful endogenous markers to identify the proliferating germinative cells in metacestode tissues, and open the possibility to identify such cells in *in vivo* material (in the absence of labeling with thymidine analogs). Despite the morphological uniformity of germinative cells, gene expression analyses have clearly shown that the germinative cells are heterogeneous at the molecular level: *em-ago-2*, *em-nos-1* and *em-nos-2* genes are only expressed in subpopulations of the proliferating germinative cells. Because Argonaute and Nanos proteins are well known post-transcriptional regulators with important roles in stem cell and germ cell biology [11,19,85], this points to the existence of different sub-populations of germinative cells, perhaps with different self-renewal or differentiation potencies.

The capacity for self-renewal of at least some of the germinative cells is strongly suggested by the HU-mediated depletion experiments, and is expected given the ability of metacestode tissue to be indefinitely passaged *in vivo*[34,35]. It is interesting that no proliferation response was observed after milder depletions, and the metacestode vesicles were able to survive for long periods under these conditions. This suggests that the control of cell proliferation is relatively lax in *E. multilocularis* metacestodes. It is possible that a relatively low number of proliferating germinative cells are enough for basal tissue turnover, but that a larger number is required for actively growing vesicles. Similarly, large differences in the number of EdU incorporating cells can be observed in vesicles incubated *ex vivo* from a single infected rodent (Koziol and Brehm, unpublished data).

### Could cells expressing *nanos* in *Echinococcus* metacestodes represent the germ line?

In many free living flatworms, it is traditionally thought that the germ line is segregated by epigenesis in the juveniles or adults [12]. In fact, the limit between the somatic stem cells and the germ line is fluid in planarians and *Macrostomum*, since the somatic neoblasts are able to contribute to the germ line during regeneration [74,86,87]. However, recent studies using molecular markers have allowed the identification of germ cells already at the time of hatching, suggesting that in these flatworms the germ line may be segregated earlier than was previously thought [74,86]. For example *nanos*, another classical germ line marker, has been shown to be expressed only in the planarian germ line stem cells, but not in the morphologically indistinguishable somatic neoblasts [86-88].

In cestodes, it is assumed that the germ line originates by epigenesis, from the germinative cells in the neck region of the developing adult [12,21,89]. It is conceivable, however, that a sub-population of the germinative cells could be segregated into the germ line earlier in development, particularly since lineage tracing has never been achieved in cestode embryos [29]. In the case of *E. multilocularis*, the extensive asexual reproduction and growth in the intermediate host makes an epigenetic mechanism very likely, since early segregation of the germ line by preformation would require incorporation of cells from a segregated germ line within every new vesicle and protoscolex that is asexually generated. Our data suggests that the germinative cell sub-population expressing *nanos* homologs are not germ line cells: no expression was observed of *em-nos-1* during brood capsule and protoscolex development, and *em-nos-2* was not consistently observed in brood capsule buds. Moreover, *em-nos-2* expression in late protoscolex development suggests a role in the formation of the nervous system. Relatedly, specific *nanos* paralogs are expressed in the nervous system in many metazoans [90-93].

### *Echinococcus* primary cells as an experimental model for stem cell research

A great advantage of the *E. multilocularis* model is the possibility of long term *in vitro* culture for primary cell preparations, resulting in complete vesicle regeneration [36]. Here, we show that primary cell cultures are enriched for germinative cells, which actively proliferate from the earliest stages of development. Genes enriched in transcriptomic studies of early primary cells and depleted in HU-treated metacestodes could therefore be mined to search for germinative cell-specific expression.

The mechanism for enrichment is not clear, and could involve differential extraction of the germinative cells from the metacestode tissues, differential survival and aggregation of the germinative cells during culture, and the accumulation of germinative cells from self-renewing divisions. There is indirect evidence supporting all of these mechanisms, since: 1) it has been shown previously that 30% of all cells in fresh primary cell preparations are in S and G2/M, making the total percentage of proliferating cells very large before they are set into culture [36], 2) there is evidence of abundant cell death in electron microscopical studies and cell suspensions, and 3) germinative cells actively proliferate in the early aggregates. Besides the germinative cells, differentiated cells such as nerve cells and muscle cells are present in the aggregates. Their role in regeneration, if any, is unknown, but the neuromuscular system has been suggested to influence cell proliferation, differentiation and pattern formation in free living flatworms [94-96].

### Differences between the *Echinococcus* germinative cells and the neoblasts in other flatworms

The lack of chromatoid bodies and the absence of *vasa* and *piwi* orthologs imply important differences between the germinative cells of cestodes and the neoblasts of planarians. Furthermore, genes that are neoblast-specific in planarians, such as *hdac1* and *phb1*, are widely expressed in *E. multilocularis*. Homologs of these genes have been shown to be important for stem cell biology and cell proliferation in other organisms, but they do not have a stem cell or even a proliferating cell-specific expression in these models [67,97-99]. It is therefore possible that the specific expression of these genes in neoblasts is a planarian novelty, which may not be shared with other flatworms. At the functional level, the response of *E. multilocularis* to partial germinative cell depletion is also different to that described in free living flatworms, since in planarians and in *Macrostomum* there is always a quick response by which the neoblast population is restored to normal levels within narrow margins, even when relatively large numbers of neoblasts remain in the tissues [100,101].

Recently, Newmark and colleagues demonstrated the existence of neoblast-like cells in the trematode *Schistosoma mansoni*, and in this organism, which also lacks *vasa* and *piwi* orthologs, paralogs of these genes are specifically expressed in the neoblast-like stem cells and have important roles in their maintenance [62,102]. In *Schistosoma mansoni* sporocysts the *ago2-1* gene (an ortholog of the *em-ago2* genes) is expressed in all neoblast-like cells, and *nanos-2* is expressed in a large sub-population of neoblast-like cells. In adults, both genes are expressed in many, if not all, somatic stem cells. Therefore, differences can also be observed in the gene expression repertoire between *E. multilocularis* germinative cells and the *Schistosoma* neoblast-like cells, making the *E. multilocularis* stem cell system unique among flatworms. Further gene expression studies will depict a clearer picture of their similarities and differences, including the analysis of *vasa*-like genes (paralogs of *vasa* such as *PL-10*) which are expressed in planarian and schistosome stem cells [13,18,102].

The *E. multilocularis* metacestode, with its ability to grow infiltratively like a tumor into the tissues of the host, is a relatively recent evolutionary novelty and is derived from the more typical cysterci larvae found in other taeniid cestodes [103,104]. Although rare in most cestodes, asexual reproduction by the formation of new scoleces is found in many taeniids [103,105-107] and it is possible that the huge proliferative potential of *E. multilocularis* germinative cells and larvae was adapted from an already increased potential found in a common taeniid ancestor. The germinative cells in *E. multilocularis*, although similar in morphology and functionally analogous to the neoblasts in other flatworms, show important differences at the level of gene expression, which could be related to this recent evolutionary change in their developmental biology. At this point, nothing is known about gene expression in the germinative cells of adult cestodes. Because of the biological hazard related to working with *E. multilocularis* adults, comparative studies in related cestode models such as *Hymenolepis microstoma*[108] and *Mesocestoides corti*[24,109] would be of great importance to delineate which characteristics of the germinative cells of *E. multilocularis* metacestodes are unique to this species and larval form, and which are a general feature of cestode germinative cells.

## Conclusions

*E. multilocularis* is an important model for the study of parasite development and host-parasite interactions, and is the only flatworm model with a robust cell culture system [110]. Growth and regeneration of *E. multiloculari*s larvae is driven by the germinative cells, which are morphologically and functionally similar to the neoblasts of free living flatworms, but which show important differences in their gene expression patterns and in the loss of conserved stem cell regulators. This work represent the first description of the germinative cells at the molecular level, giving the first evidence of the existence of sub-populations of germinative cells with different gene expression patterns, and provides molecular markers for identifying differentiated cell types *in situ.* Some differences between the germinative cells and the neoblasts of other flatworms could have arisen as specific adaptations of the stem cell system for the unique asexual development of *E. multilocularis*. The novel development of *E. multilocularis* and other taeniids is therefore an excellent model for the study of the evolutionary origins of asexual reproduction and its effect on stem cell systems.

## Abbreviations

AcTub: acetylated tubulin alpha; bc: brood capsule; BrdU: 5-bromo-2′-deoxyuridine; CHAPS: 3-[(3-cholamidopropyl)dimethylammonio]-1-propanesulfonate; CDS: coding domain sequence; DAPI: 4′, 6-diamidino-2-phenylindole; DEPC: diethyl pyrocarbonate; dNTPs: deoxyribonucleotide triphosphates; EDTA: ethylenediaminetetraacetic acid; EdU: 5-ethynyl-2′-deoxyuridine; ELISA: Enzyme-Linked Immunosorbent Assay; FITC: fluorescein isothiocyanate; GL: germinal layer; H3S10-P: histone H3 phosphorylated in Serine 10; HF: hydatid fluid; HMW: high molecular weight; HU: hydroxyurea; LL: laminated layer; MAB: maleic acid buffer; NBT/BCIP: nitro blue tetrazolium chloride and 5-bromo-4-chloro-3-indolyl phosphate; NR: Nile red; PBS: phosphate buffer saline; PCR: polymerase chain reaction; PFA: paraformaldehyde; PI: propidium iodide; PS: protoscolex; RNP: ribonucleoprotein; RRM: ribonucleotide reductase; RT-PCR: reverse transcription polymerase chain reaction; SSC: saline-sodium citrate buffer; TEA: triethanolamine; TEM: transmission electron microscopy; WCS: Whole Cell Stain; WMISH: whole-mount *in situ* hybridization.

## Competing interests

The authors declare that they have no competing interests.

## Authors’ contributions

UK: contributed to study design, carried out or participated in all experiments, analyzed and interpreted the data, manuscript writing and final approval of the manuscript. KB: designed the study, analyzed and interpreted the data, carried out critical revision and final approval of the manuscript. GK: data collection and interpretation and final approval of the manuscript. TR: data collection and analysis and final approval of the manuscript. LZ: data collection and analysis and final approval of the manuscript. All authors read and approved the final manuscript.

## Supplementary Material

Additional file 1 List of GeneDB gene codes, primers and probes from this work. Click here for file

Additional file 2 Representative examples of WMISH experiments and the respective controls performed with sense probes, for genes with different levels of expression. Click here for file

Additional file 3 **Example of a rare mitotic EdU + cell after a 50 μM five hour pulse.** DAPI staining is shown in blue, EdU detection in red, and tubulin immunohistofluorescence in green. The bar represents 5 μm. Click here for file

Additional file 4 **EdU incorporation in *ex vivo *cultured metacestode material.** Abbreviations: bc, brood capsule; dev ps, developing protoscoleces; gl, germinal layer; inv ps, invaginated protoscolex; host, host tissue. Bars represent 200 μm. Click here for file

Additional file 5 **EdU incorporation after protoscolex isolation and activation.** The protocol and representative images of EdU incorporation are shown for each condition. The experiment was repeated twice with similar results. Click here for file

Additional file 6 **Histogram of cell areas as seen in cell suspensions (used as a proxy for cell size) for all germinative cells and for EdU + germinative cells in the germinal layer and in primary cell preparations.** Smaller cells are less likely to incorporate EdU, and EdU + cells are overrepresented at intermediate sizes. This is compatible with smaller cells being in G1/G0-phase, cells of intermediate size in S-phase and the larger cells in G2-phase. However, it is possible that other factors (such as different germinative cell sub-populations) also affect germinative cell size. Click here for file

Additional file 7 **Example of a EdU + germinative cell in a cell suspension prepared from protoscoleces (previously incubated for five hours in 50 μM EdU).** WCS is shown in green, EdU in red and DAPI in blue. Click here for file

Additional file 8 **Genomic organization of *em-ago2 *genes.** (A) Graphical representation of the end of scaffold 7767 and the beginning of scaffold 7771, showing the position of primers used for PCR with genomic DNA. Both DNA strands and all six reading frames are shown, together with the position of genes and their exons and the *em-ago2-*ψ pseudogene. (B) PCR with genomic DNA to confirm the structure of both scaffolds. Lanes 1, 2 and 3 in each gel indicate primer sets 1 F/R, 2 F/R, 3 F/R for scaffolds 7767 and 7771, and lane 4 indicates primer set 4 F/R for scaffold 7771. Bands of the expected size are observed in all cases (indicated by red dots) except for the primer combination 7767-3 F/R. It is possible that a difference occurs in the analyzed isolate (MP1) as compared to the reference genome in this particular region. Click here for file

Additional file 9 **Fluorescent WMISH of *em-hdac1 *** **on metacestode vesicles. Bars represent 40 μm.** Click here for file

Additional file 10 **WMISH of *em-phb1 *** **on metacestode vesicles, on the germinal layer (upper panel), brood capsules (middle panel) and protoscolex buds (lower panel).** Click here for file

Additional file 11 **Immunohistofluorescence of PHB1 in sections of *Dugesia tahitiensis*.** (A) General view of a cross section at the level of the pharynx (DAPI stained). (A1 and A2) Detail of the region of the pharynx (f) and the nerve cords (Figure A1) and of the mesenchyme surrounding the intestine (i) (Figure A2), as seen with DAPI and PHB1 immunoreactivity. Because of strong auto-fluorescence seen in the region of the gut and epidermis in all fluorescence channels, we also provide the signal in the rhodamine channel for comparison (auto-fluorescence thus appears yellow in the merged image). PHB1 is most strongly expressed in small neoblast-like cells in the mesenchyme and around the nerve cords. (B) Negative control (no primary antibody). Bars represent 50 μm in A1, A2 and B, and 20 μm in the detailed views. Click here for file

Additional file 12 **Em-PHB1 immunodetection in *E. multilocularis*.**Top, immunohistochemistry (IHC) of PHB1 in sections of a vesicle with brood capsules and protoscoleces. Bottom, control IHC without any primary antibody. Bars represent 20 μm. Click here for file

Additional file 13 **Details of alkaline phosphatase activity in *E. multilocularis*.** (A) Lack of alkaline phosphatase activity in the tegument of the brood capsule. (B) Qualitative assessment of whole-mount alkaline phosphatase activity in the excretory system of activated and non-activated protoscoleces. One hundred and fifty protoscoleces were examined and their signal classified as either strong (strong signal in scolex and body), moderate (moderate signal only in scolex or body), weak (barely detectable signal) or not stained (no signal observed). Click here for file

Additional file 14 **Differentiation of nerve cells in EdU continuous labeling experiments.** (A) Double detection of AcTub immunohistofluorescence and EdU incorporation after a five hour 50 μM pulse. No double positive cells can be detected. (B) Example of a double positive cell after 14 days of incubation in 10 μM EdU. (C) Percentage of AcTub + EdU + double positive cells over all AcTub + cells after five hours, seven days and fourteen days of incubation in medium containing 1 μM or 10 μM EdU (average and standard deviation of two to three metacestode vesicles per time point and condition). Bars represent 20 μm. Click here for file

Additional file 15 **Immunohistofluorescence with anti-HMW-tropomyosin in metacestode sections.** (A) Germinal layer. (B) Brood capsule. (C) Brood capsule with a protoscolex bud. (D) Invaginated protoscolex. Arrowheads point to the subtegumental muscle layer, which is greatly thickened in brood capsules as compared to the germinal layer. Bars represent 20 μm. Click here for file

Additional file 16 **Effect of hydroxyurea (HU) on primary cells.** (A) Effect on proliferation as assayed by BrdU incorporation and ELISA detection. The BrdU incorporation levels in non-treated controls was set as 100% and relative values are shown for 10 mM and 40 mM HU, as well as for the control without BrdU (average and standard deviation of four independent experiments). (B) Effect on new vesicle of regeneration from primary cells after three weeks of culture (average and standard deviation of two independent experiments). Click here for file

Additional file 17 Example of degenerating cell in TEM analysis of early (two days of culture) primary cell preparations. Click here for file

Additional file 18 **AcTub immunohistofluorescence and phalloidin staining of primary cell aggregates after three days of culture.** Bars represent 20 μm. Click here for file
