# Supplementary figures and images for: The unique stem cell system of the immortal larva of the human parasite Echinococcus multilocularis
Source: EvoDevo. 2014 Mar 6;5:10. doi: 10.1186/2041-9139-5-10 (PMC4015340; doi:10.1186/2041-9139-5-10)

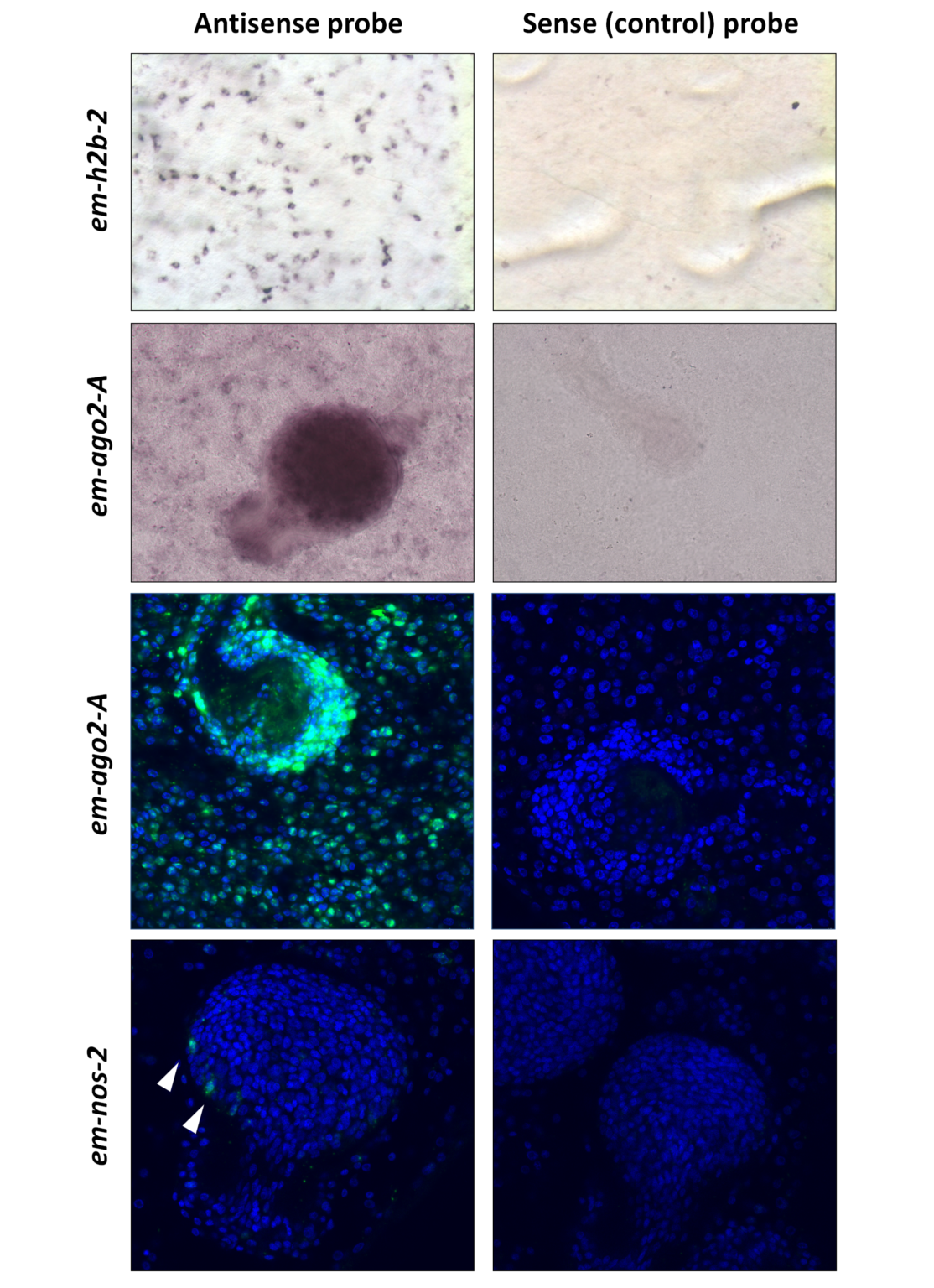

Supplement: Additional file 2 — Representative examples of WMISH experiments and the respective controls performed with sense probes, for genes with different levels of expression. [file 2041-9139-5-10-S2.tiff]

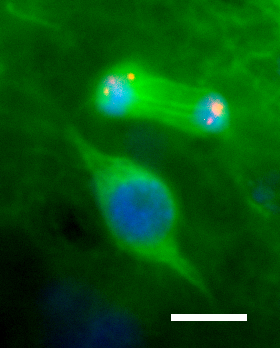

Supplement: Additional file 3 — Example of a rare mitotic EdU + cell after a 50 μM five hour pulse. DAPI staining is shown in blue, EdU detection in red, and tubulin immunohistofluorescence in green. The bar represents 5 μm. [file 2041-9139-5-10-S3.tiff]

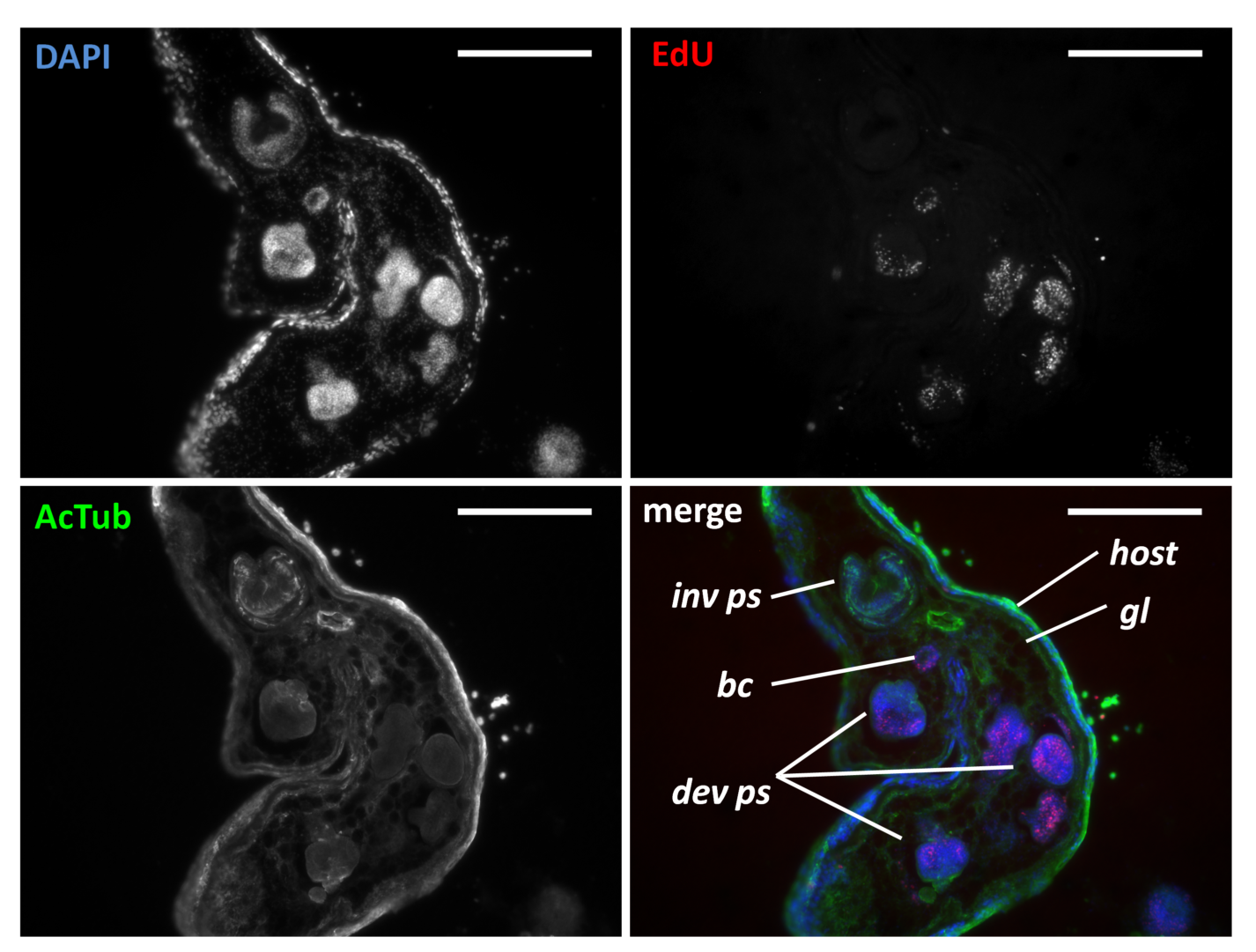

Supplement: Additional file 4 — EdU incorporation in ex vivo cultured metacestode material. Abbreviations: bc, brood capsule; dev ps, developing protoscoleces; gl, germinal layer; inv ps, invaginated protoscolex; host, host tissue. Bars represent 200 μm. [file 2041-9139-5-10-S4.tiff]

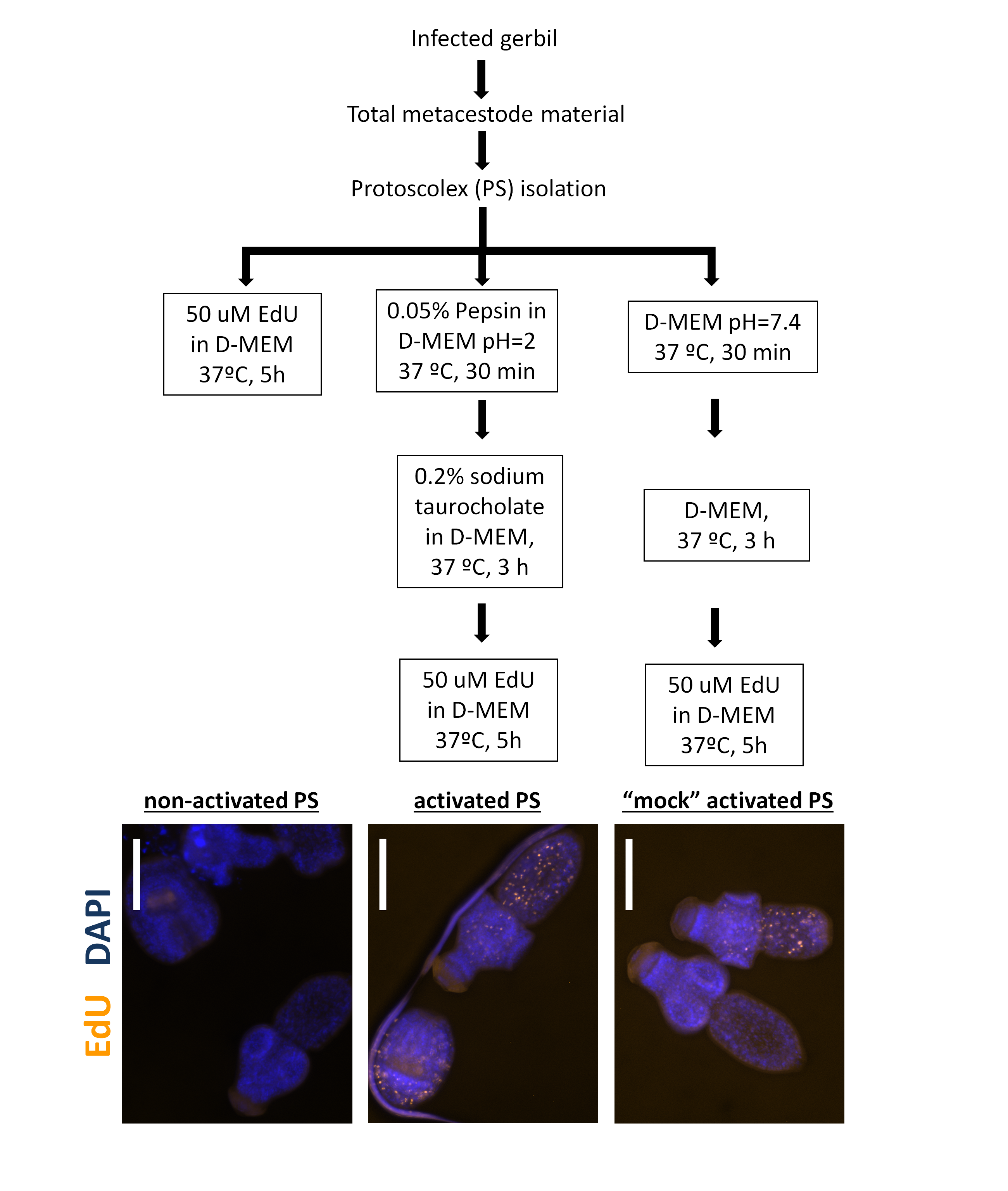

Supplement: Additional file 5 — EdU incorporation after protoscolex isolation and activation. The protocol and representative images of EdU incorporation are shown for each condition. The experiment was repeated twice with similar results. [file 2041-9139-5-10-S5.tiff]

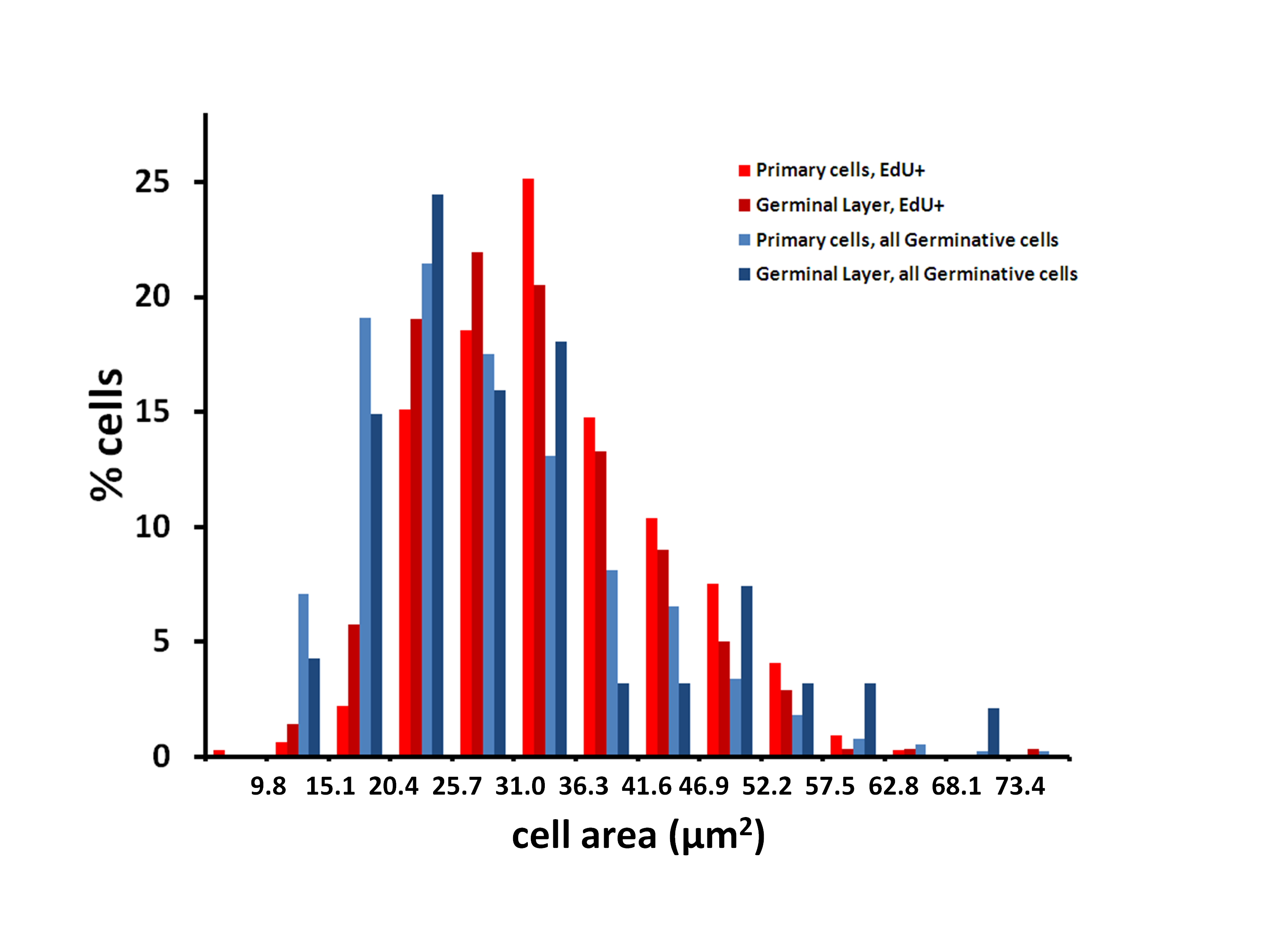

Supplement: Additional file 6 — Histogram of cell areas as seen in cell suspensions (used as a proxy for cell size) for all germinative cells and for EdU + germinative cells in the germinal layer and in primary cell preparations. Smaller cells are less likely to incorporate EdU, and EdU + cells are overrepresented at intermediate sizes. This is compatible with smaller cells being in G1/G0-phase, cells of intermediate size in S-phase and the larger cells in G2-phase. However, it is possible that other factors (such as different germinative cell sub-populations) also affect germinative cell size. [file 2041-9139-5-10-S6.tiff]

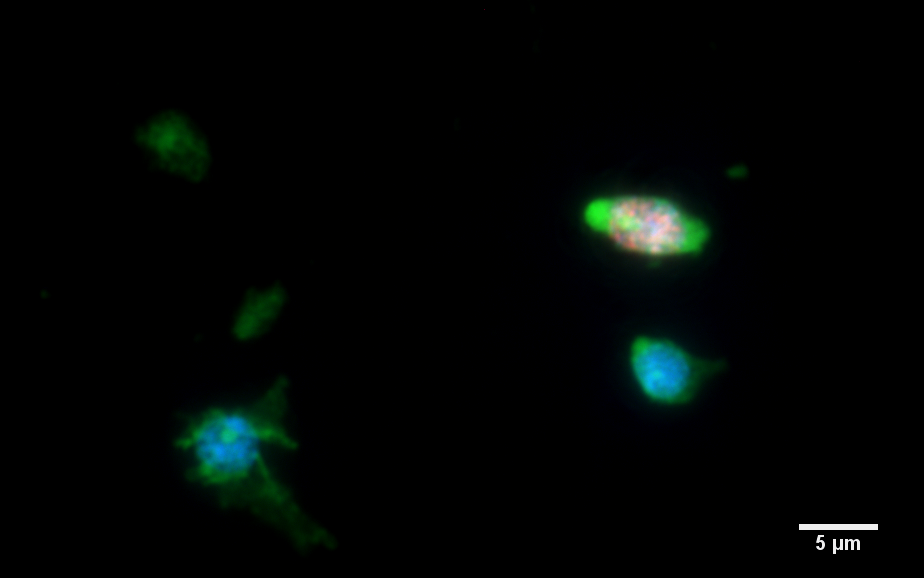

Supplement: Additional file 7 — Example of a EdU + germinative cell in a cell suspension prepared from protoscoleces (previously incubated for five hours in 50 μM EdU). WCS is shown in green, EdU in red and DAPI in blue. [file 2041-9139-5-10-S7.tiff]

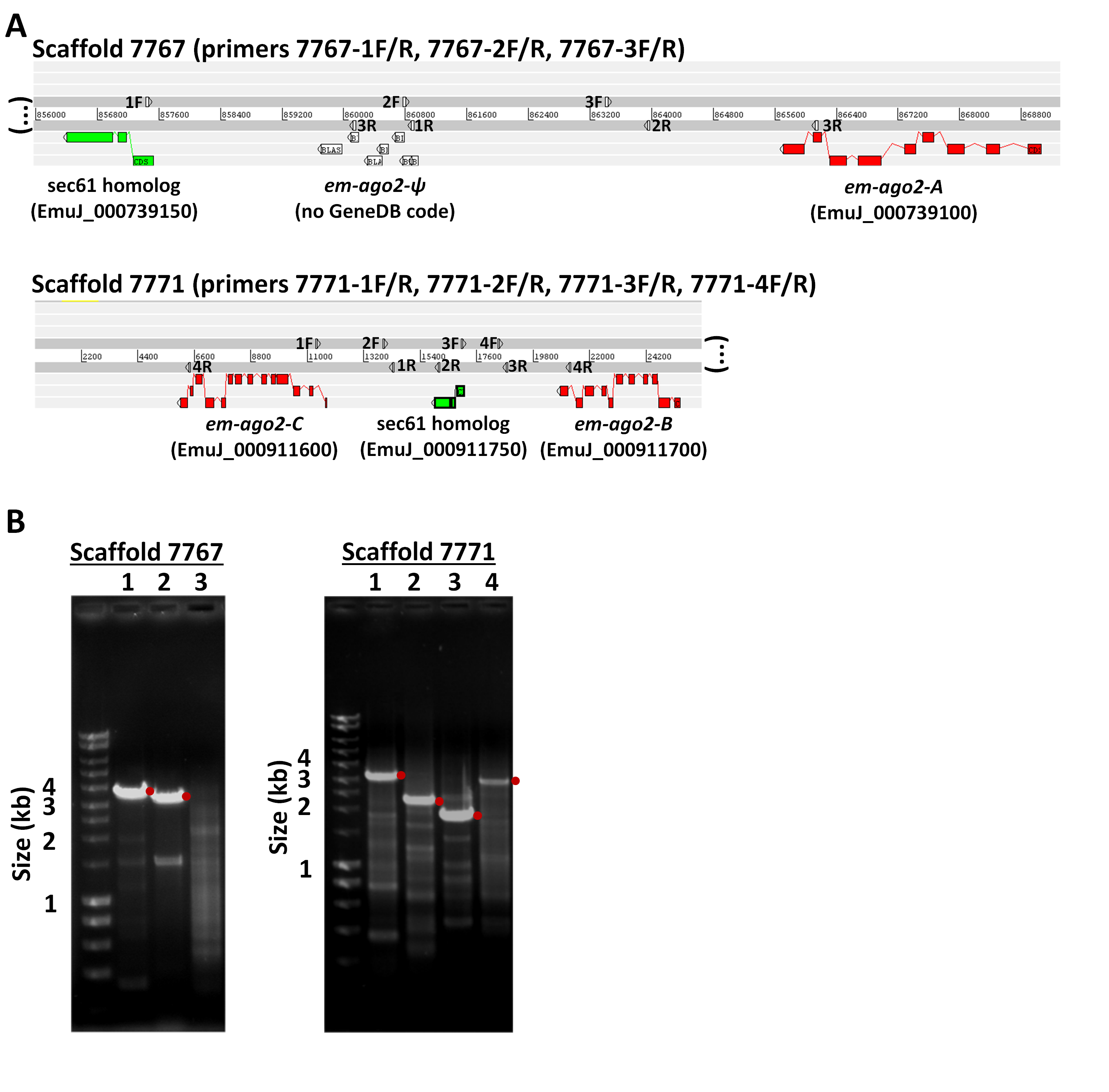

Supplement: Additional file 8 — Genomic organization of em-ago2 genes. (A) Graphical representation of the end of scaffold 7767 and the beginning of scaffold 7771, showing the position of primers used for PCR with genomic DNA. Both DNA strands and all six reading frames are shown, together with the position of genes and their exons and the em-ago2-ψ pseudogene. (B) PCR with genomic DNA to confirm the structure of both scaffolds. Lanes 1, 2 and 3 in each gel indicate primer sets 1 F/R, 2 F/R, 3 F/R for scaffolds 7767 and 7771, and lane 4 indicates primer set 4 F/R for scaffold 7771. Bands of the expected size are observed in all cases (indicated by red dots) except for the primer combination 7767-3 F/R. It is possible that a difference occurs in the analyzed isolate (MP1) as compared to the reference genome in this particular region. [file 2041-9139-5-10-S8.tiff]

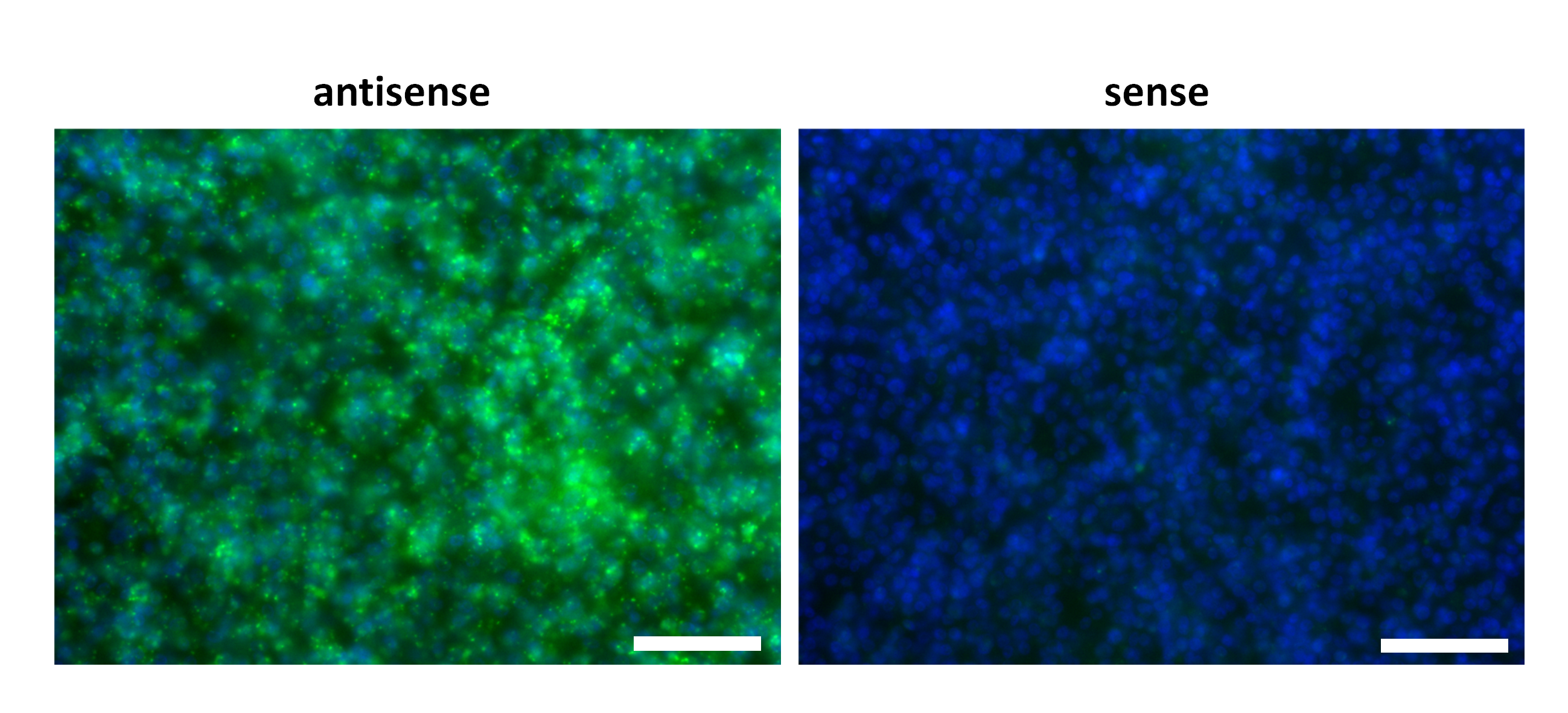

Supplement: Additional file 9 — Fluorescent WMISH of em-hdac1 on metacestode vesicles. Bars represent 40 μm. [file 2041-9139-5-10-S9.tiff]

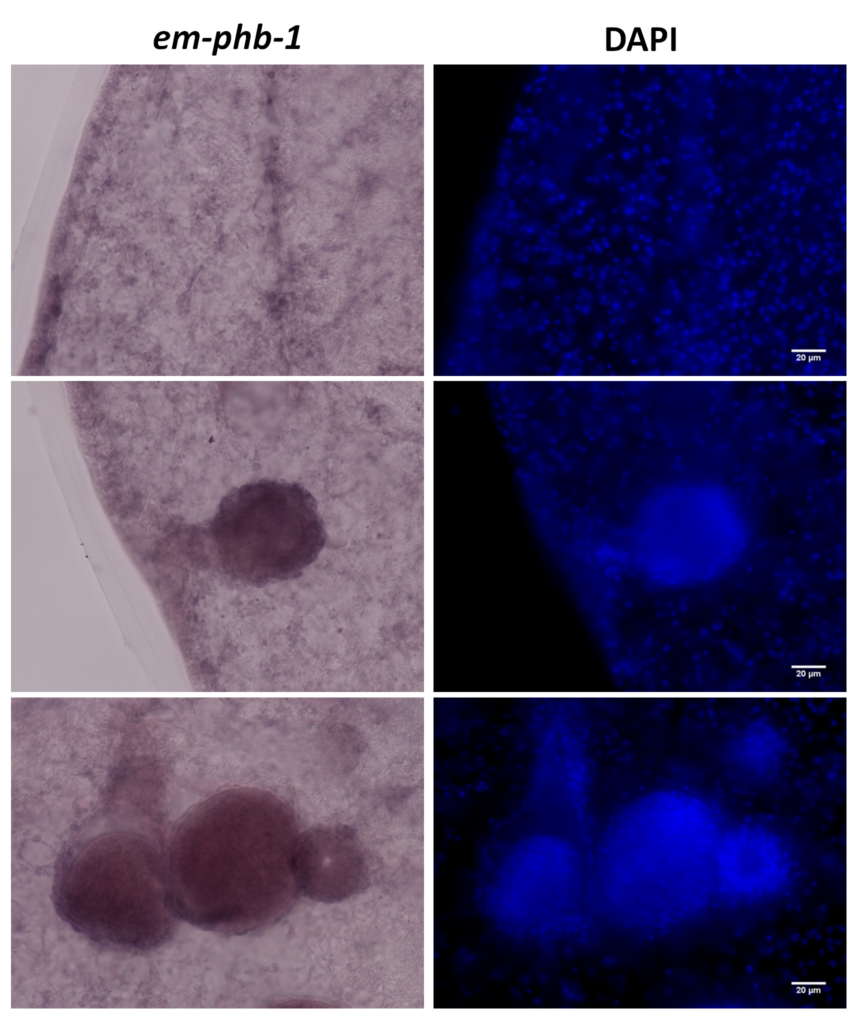

Supplement: Additional file 10 — WMISH of em-phb1 on metacestode vesicles, on the germinal layer (upper panel), brood capsules (middle panel) and protoscolex buds (lower panel). [file 2041-9139-5-10-S10.tiff]

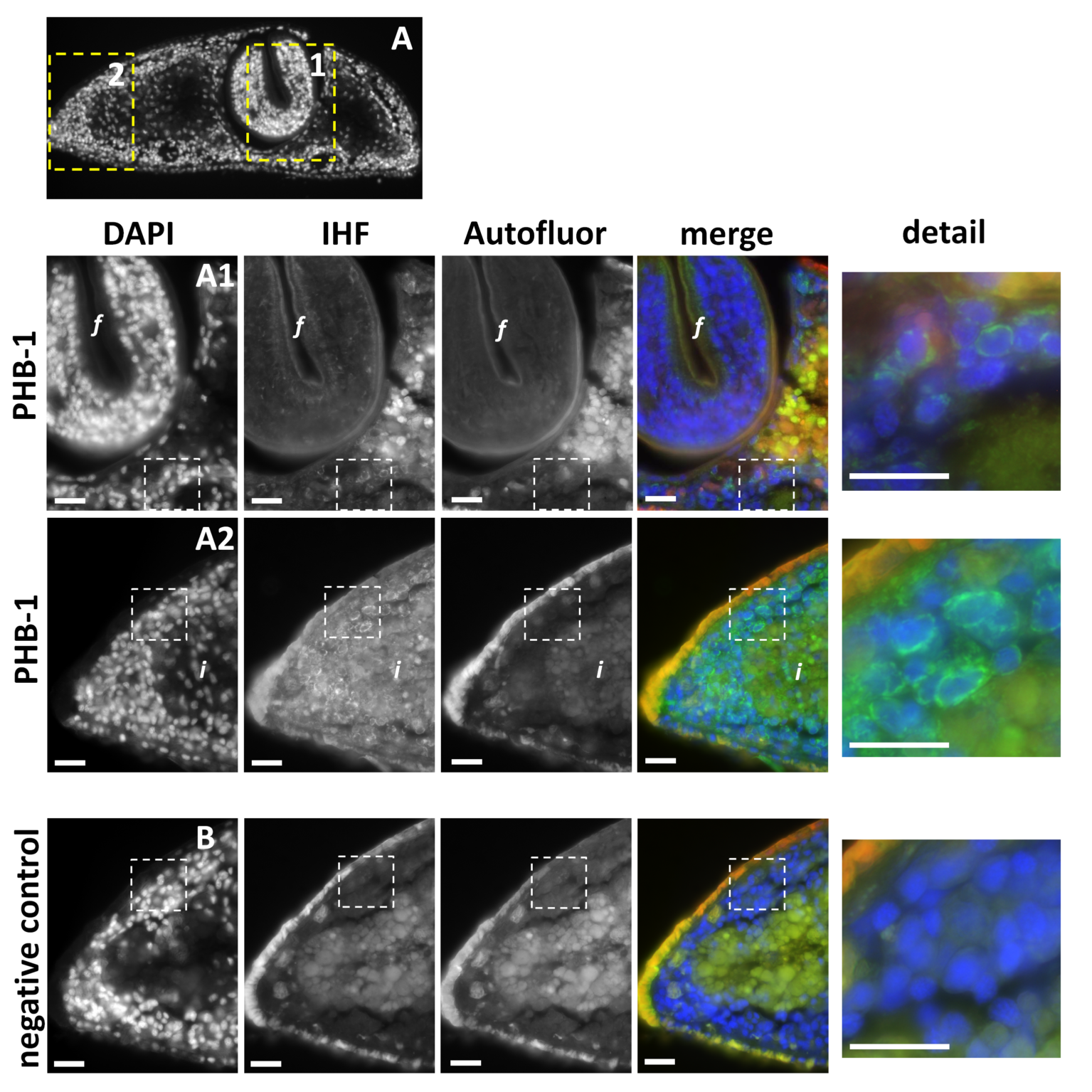

Supplement: Additional file 11 — Immunohistofluorescence of PHB1 in sections of Dugesia tahitiensis. (A) General view of a cross section at the level of the pharynx (DAPI stained). (A1 and A2) Detail of the region of the pharynx (f) and the nerve cords (Figure A1) and of the mesenchyme surrounding the intestine (i) (Figure A2), as seen with DAPI and PHB1 immunoreactivity. Because of strong auto-fluorescence seen in the region of the gut and epidermis in all fluorescence channels, we also provide the signal in the rhodamine channel for comparison (auto-fluorescence thus appears yellow in the merged image). PHB1 is most strongly expressed in small neoblast-like cells in the mesenchyme and around the nerve cords. (B) Negative control (no primary antibody). Bars represent 50 μm in A1, A2 and B, and 20 μm in the detailed views. [file 2041-9139-5-10-S11.tiff]

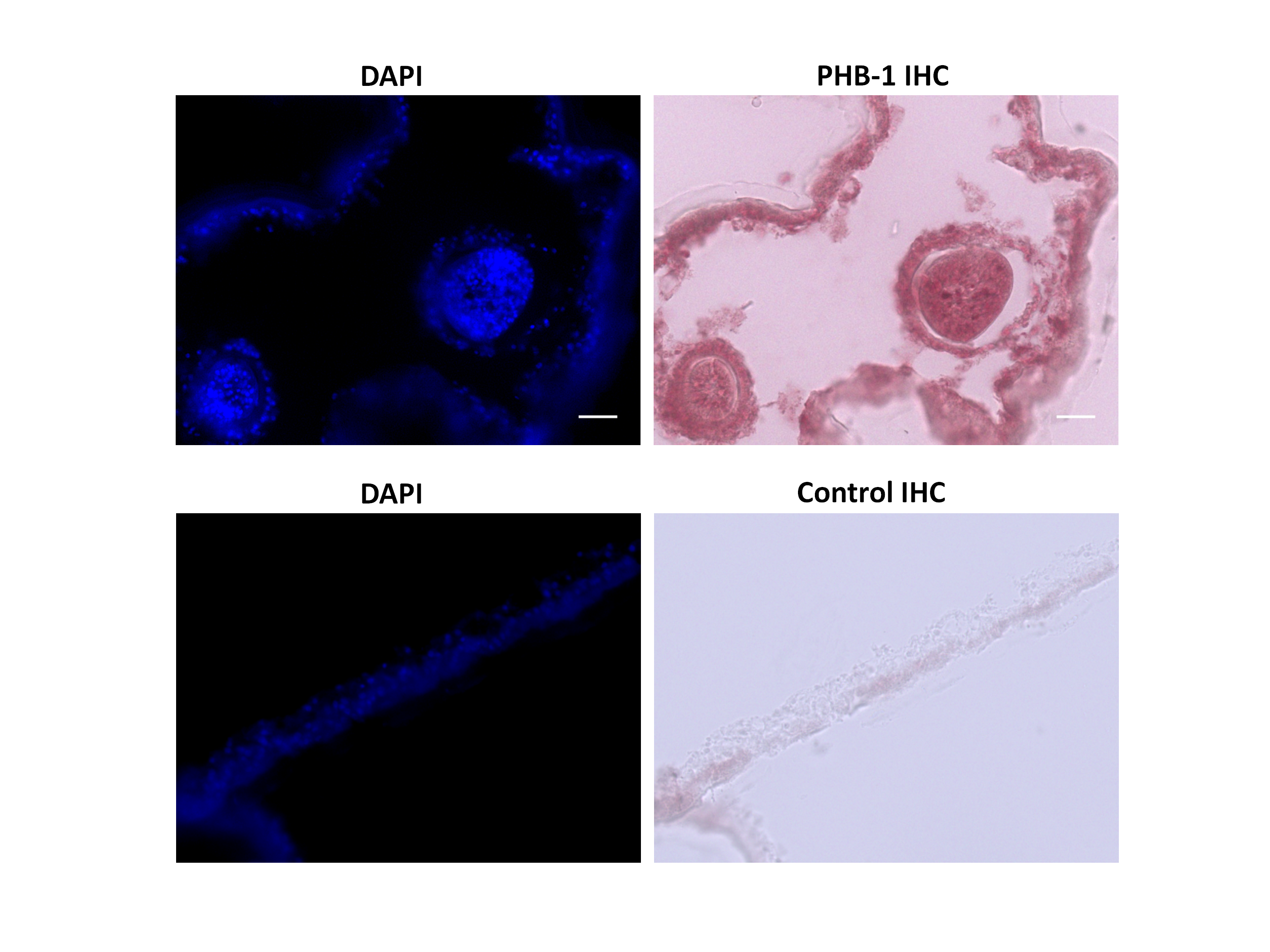

Supplement: Additional file 12 — Em-PHB1 immunodetection in E. multilocularis.Top, immunohistochemistry (IHC) of PHB1 in sections of a vesicle with brood capsules and protoscoleces. Bottom, control IHC without any primary antibody. Bars represent 20 μm. [file 2041-9139-5-10-S12.tiff]

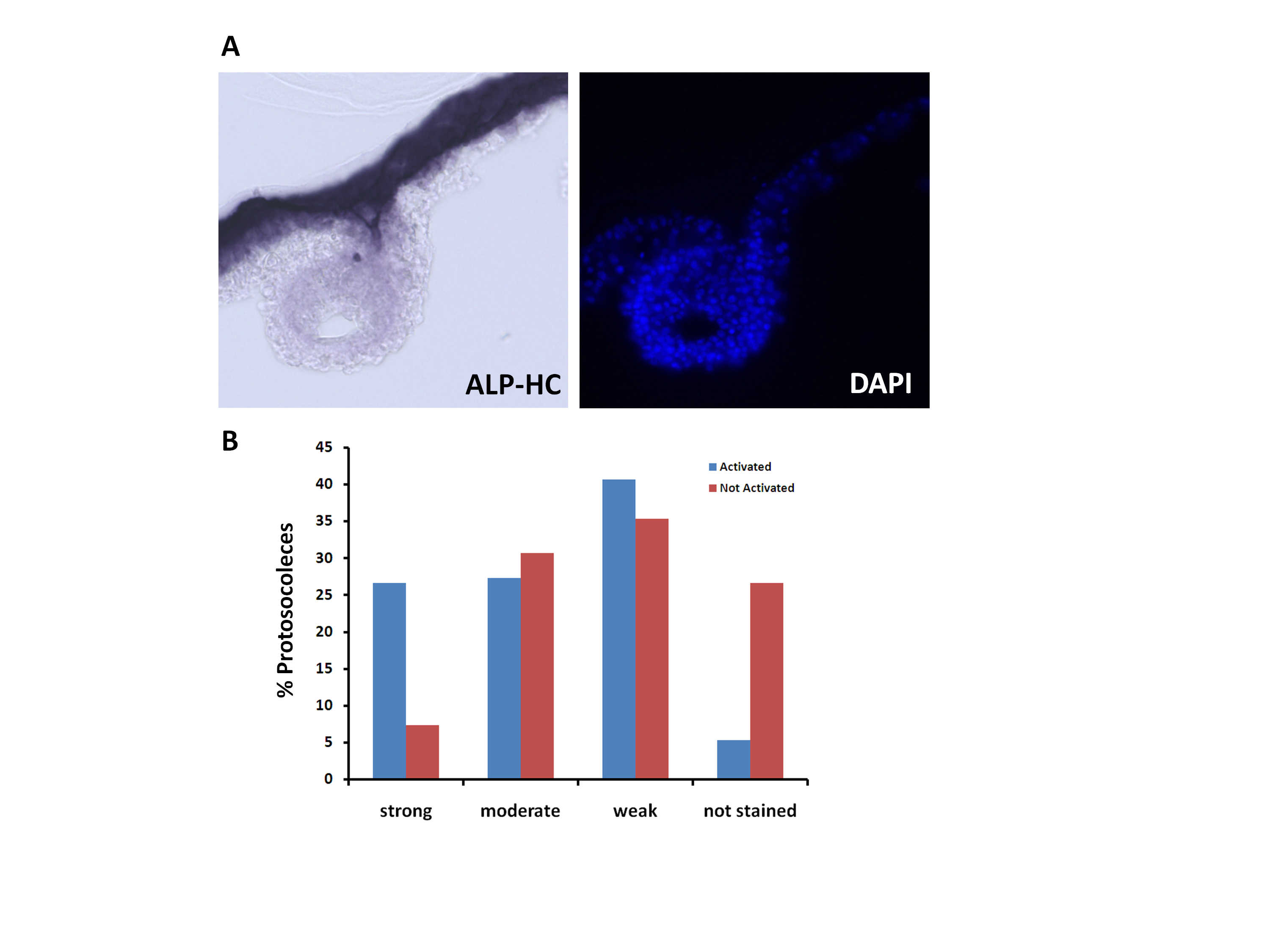

Supplement: Additional file 13 — Details of alkaline phosphatase activity in E. multilocularis. (A) Lack of alkaline phosphatase activity in the tegument of the brood capsule. (B) Qualitative assessment of whole-mount alkaline phosphatase activity in the excretory system of activated and non-activated protoscoleces. One hundred and fifty protoscoleces were examined and their signal classified as either strong (strong signal in scolex and body), moderate (moderate signal only in scolex or body), weak (barely detectable signal) or not stained (no signal observed). [file 2041-9139-5-10-S13.tiff]

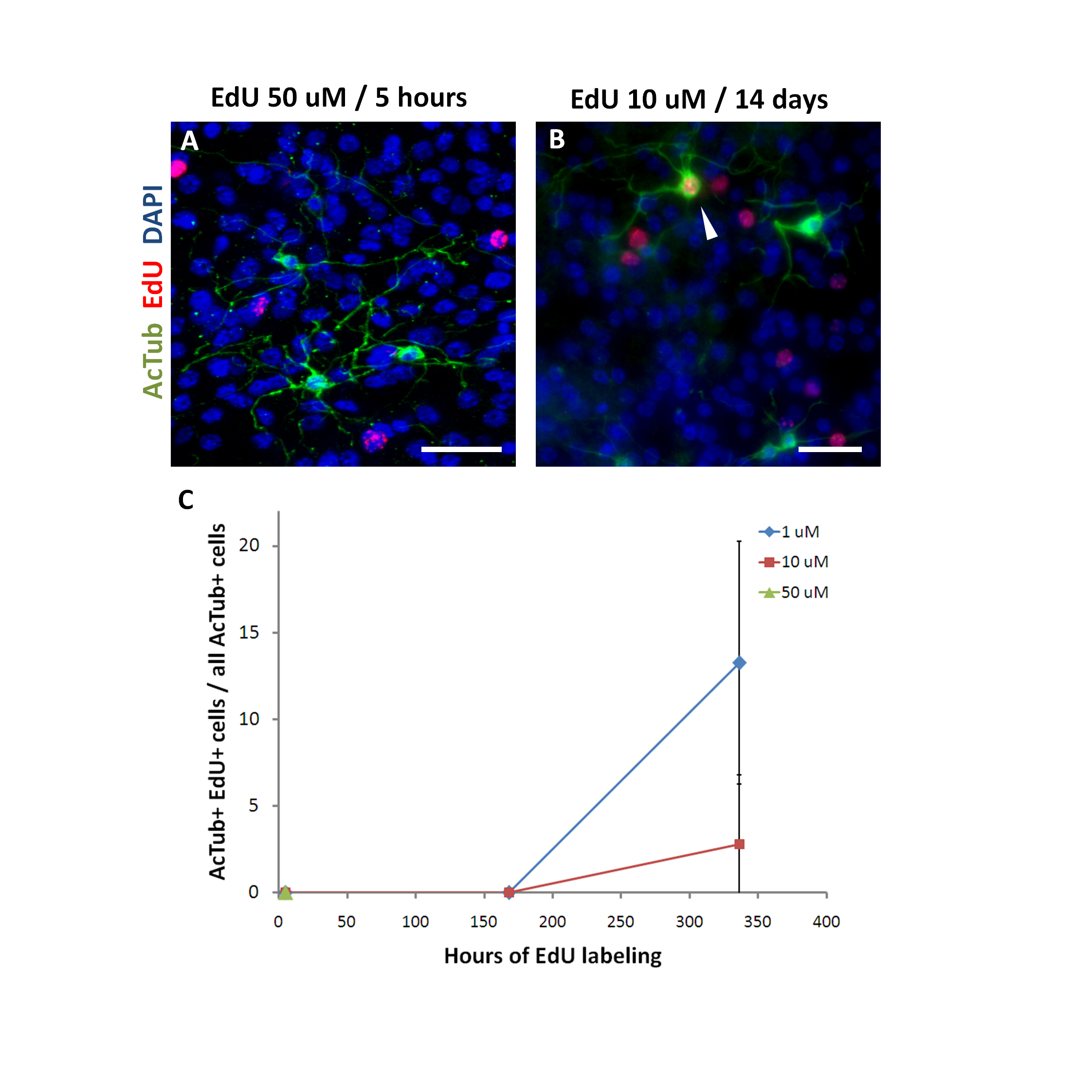

Supplement: Additional file 14 — Differentiation of nerve cells in EdU continuous labeling experiments. (A) Double detection of AcTub immunohistofluorescence and EdU incorporation after a five hour 50 μM pulse. No double positive cells can be detected. (B) Example of a double positive cell after 14 days of incubation in 10 μM EdU. (C) Percentage of AcTub + EdU + double positive cells over all AcTub + cells after five hours, seven days and fourteen days of incubation in medium containing 1 μM or 10 μM EdU (average and standard deviation of two to three metacestode vesicles per time point and condition). Bars represent 20 μm. [file 2041-9139-5-10-S14.tiff]

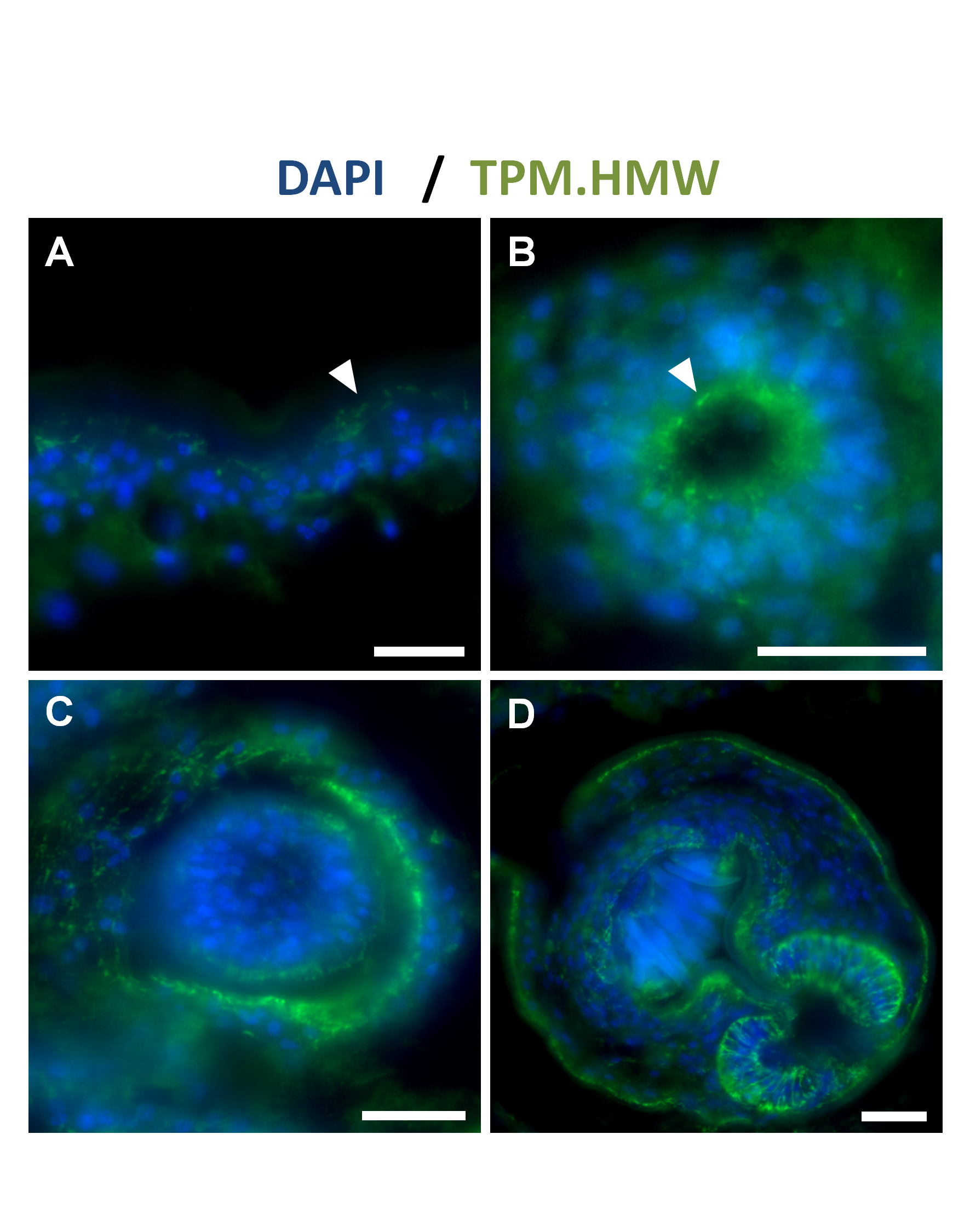

Supplement: Additional file 15 — Immunohistofluorescence with anti-HMW-tropomyosin in metacestode sections. (A) Germinal layer. (B) Brood capsule. (C) Brood capsule with a protoscolex bud. (D) Invaginated protoscolex. Arrowheads point to the subtegumental muscle layer, which is greatly thickened in brood capsules as compared to the germinal layer. Bars represent 20 μm. [file 2041-9139-5-10-S15.tiff]

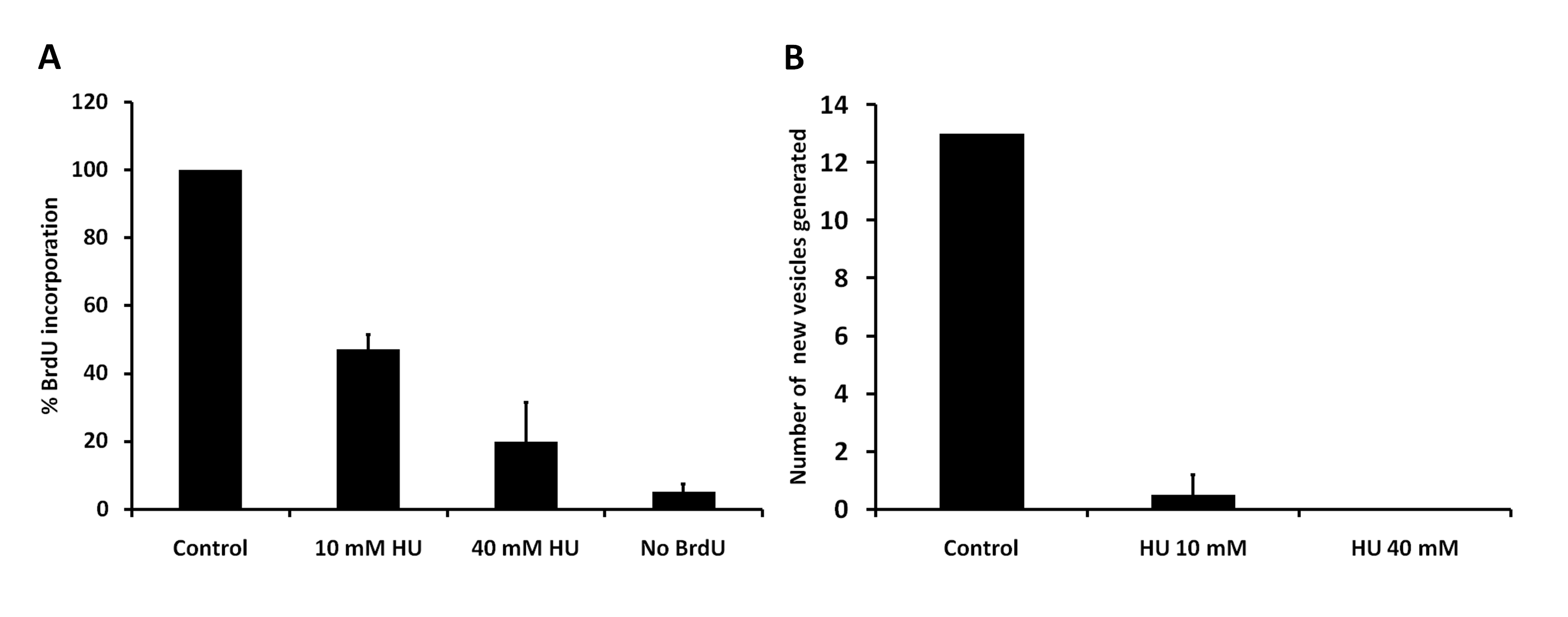

Supplement: Additional file 16 — Effect of hydroxyurea (HU) on primary cells. (A) Effect on proliferation as assayed by BrdU incorporation and ELISA detection. The BrdU incorporation levels in non-treated controls was set as 100% and relative values are shown for 10 mM and 40 mM HU, as well as for the control without BrdU (average and standard deviation of four independent experiments). (B) Effect on new vesicle of regeneration from primary cells after three weeks of culture (average and standard deviation of two independent experiments). [file 2041-9139-5-10-S16.tiff]

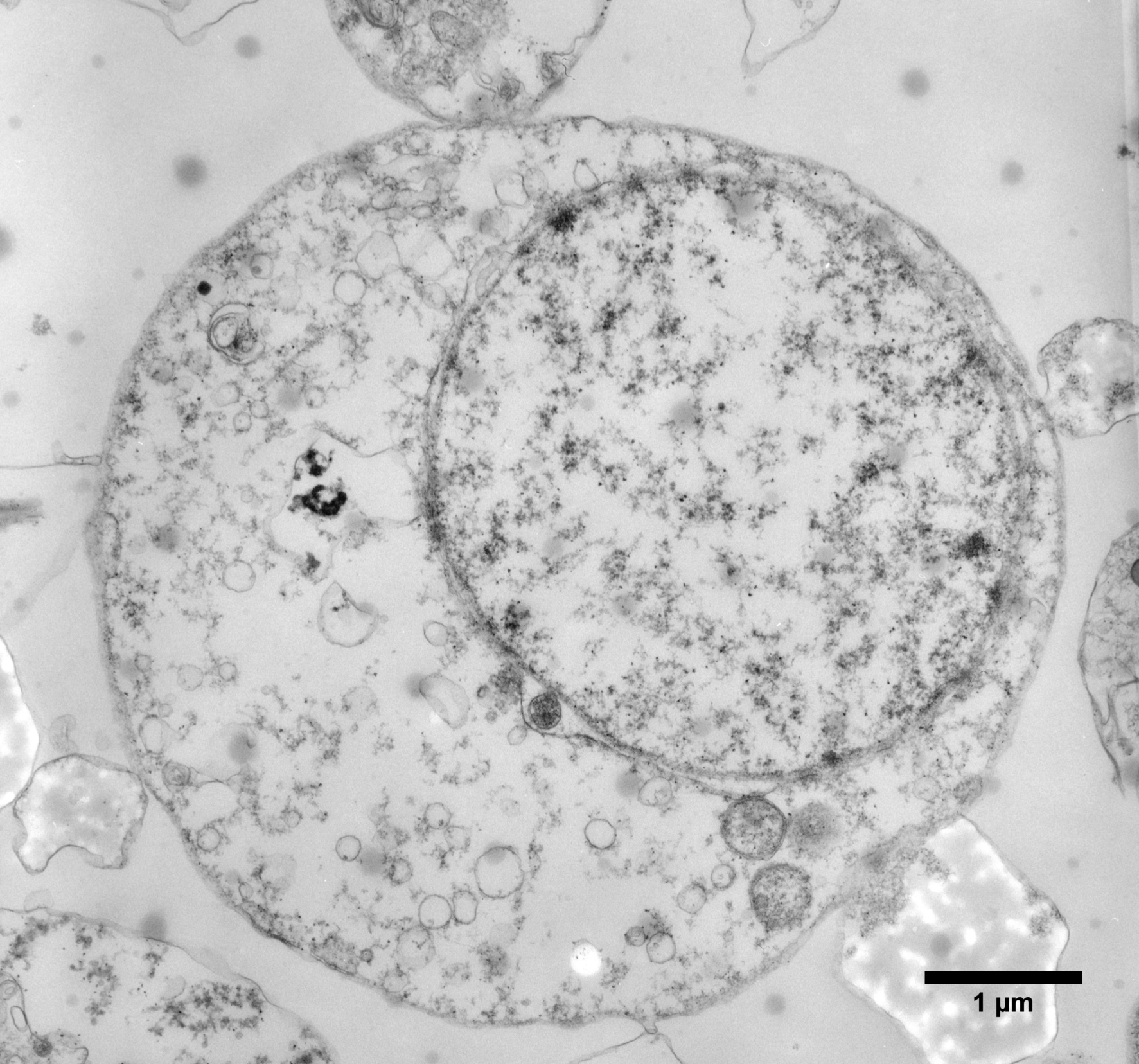

Supplement: Additional file 17 — Example of degenerating cell in TEM analysis of early (two days of culture) primary cell preparations. [file 2041-9139-5-10-S17.tiff]

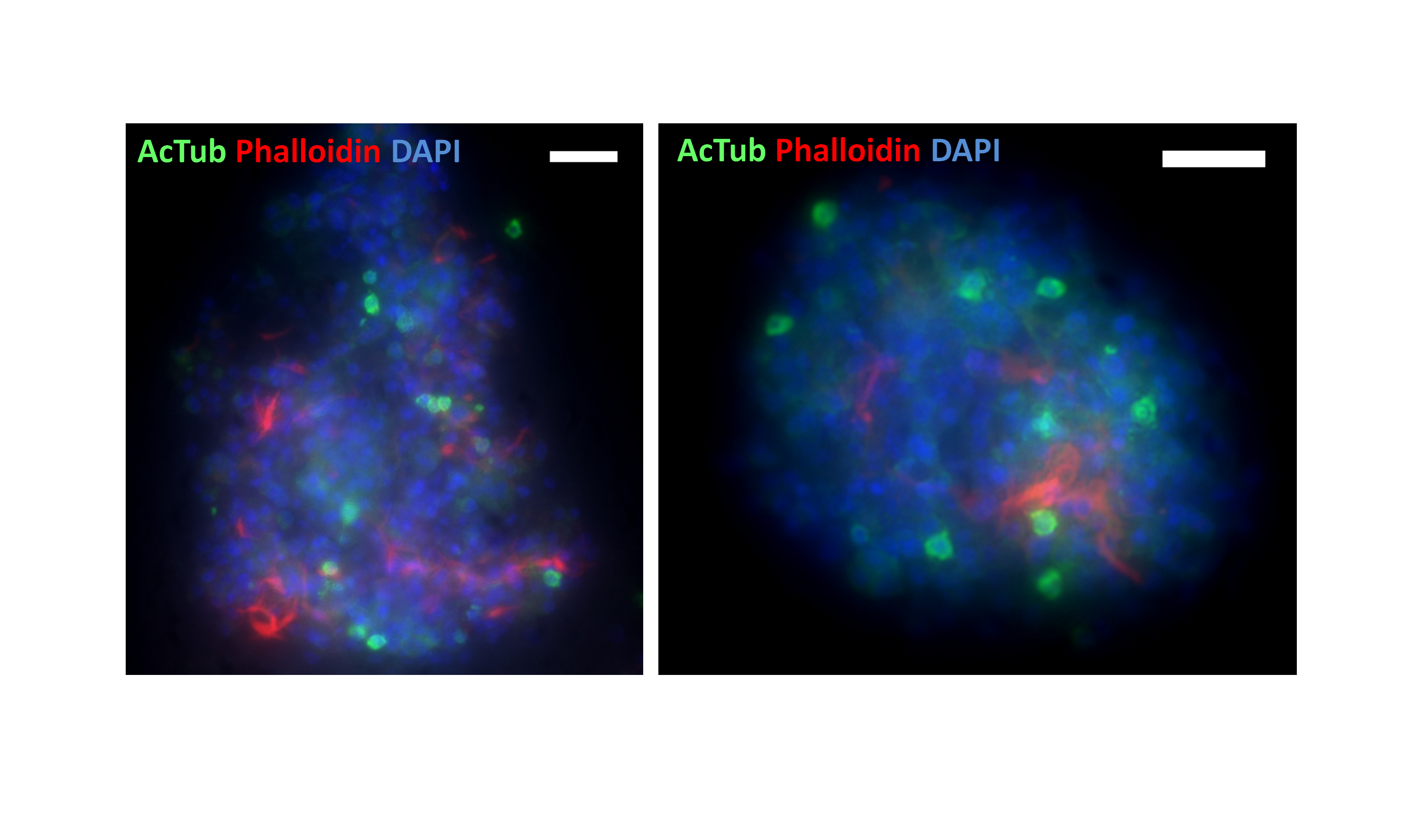

Supplement: Additional file 18 — AcTub immunohistofluorescence and phalloidin staining of primary cell aggregates after three days of culture. Bars represent 20 μm. [file 2041-9139-5-10-S18.tiff]
